# Vasomotor Symptoms and Their Association with Depressive and Anxiety Symptoms in Peri- and Postmenopausal Women: A Systematic Review of Observational Studies

**DOI:** 10.3390/medicina62081574

**Published:** 2026-08-17

**Authors:** Oana Neda-Stepan, Virgil Radu Enătescu, Catalina Giurgi-Oncu, Elena Silvia Bernad, Radu Neamțu, Brenda-Cristiana Bernad, Alin Vasile Kadaș, Cristina Bredicean

**Affiliations:** 1 Department of Neurosciences, Discipline of Psychiatry, “Victor Babeș” University of Medicine and Pharmacy, Eftimie Murgu Square 2, 300041 Timișoara, Romaniaenatescu.virgil@umft.ro (V.R.E.); catalina.giurgi@umft.ro (C.G.-O.); bredicean.ana@umft.ro (C.B.); 2 Department of Obstetrics and Gynecology, “Victor Babeș” University of Medicine and Pharmacy, Eftimie Murgu Square 2, 300041 Timișoara, Romania; bernad.elena@umft.ro (E.S.B.); radu.neamtu@umft.ro (R.N.); 3 Center for Laparoscopy, Laparoscopic Surgery and In Vitro Fertilization, Faculty of Medicine, Center for Neuropsychology and Behavioral Medicine, “Victor Babeș” University of Medicine and Pharmacy, Eftimie Murgu Square 2, 300041 Timișoara, Romania; 4 Ist Clinic of Obstetrics and Gynecology, Laparoscopy, In Vitro Fertilization and Embryotransfer Research Center, “Pius Brînzeu” County Clinical Emergency Hospital, 300723 Timișoara, Romania; 5 Doctoral School, “Victor Babeș” University of Medicine and Pharmacy, Eftimie Murgu Square 2, 300041 Timișoara, Romania; bernad.brenda@umft.ro; 6 Department of Neuroscience, Center for Neuropsychology and Behavioral Medicine, “Victor Babeș” University of Medicine and Pharmacy, Eftimie Murgu Square 2, 300041 Timișoara, Romania

**Keywords:** menopause, perimenopause, vasomotor symptoms, hot flashes, night sweats, depression, anxiety, women’s mental health, systematic review

## Abstract

*Background and Objectives*: Vasomotor symptoms (VMS), including hot flushes and night sweats, often co-occur with depressive and anxiety symptoms during the menopausal transition. This systematic review assessed the observational evidence for a quantitative association between VMS burden and affective symptoms in peri- and postmenopausal women. *Materials and Methods*: PubMed and PubMed Central were searched from database inception to 30 June 2025 for English-language, open-access observational studies reporting VMS and depressive and/or anxiety outcomes in peri- or postmenopausal women. Two reviewers independently screened records and full-text reports, and extracted data were independently verified. Study characteristics, symptom measures, prevalence or severity data, and quantitative association estimates were extracted. Owing to substantial heterogeneity of exposure and outcome measures, a structured narrative synthesis was conducted in accordance with the Synthesis Without Meta-analysis (SWiM) guideline. Risk of bias was appraised at item level using the Joanna Briggs Institute checklists and the Newcastle–Ottawa Scale, and the certainty of the evidence was rated using the GRADE approach. *Results*: Twelve studies involving approximately 9900 women from seven countries were included. In studies reporting adjusted estimates, greater VMS burden was associated with higher odds of depressive symptoms, with adjusted odds ratios ranging from 1.67 to 2.99; one longitudinal cohort reported an odds ratio of 2.95 for a worsening depressive-symptom trajectory. Evidence for anxiety was directionally concordant but considerably weaker: anxiety was assessed as a distinct outcome in only five of the 12 studies, none of which reported an adjusted effect estimate, and two of which were rated at high concern for risk of bias. Two studies were rated at overall low concern for risk of bias, seven at moderate concern, and three at high concern. Several studies suggested partial mediation through fatigue or poor sleep, whereas others supported direct or bidirectional associations. *Conclusions*: Greater VMS burden is consistently associated with depressive symptoms and, on a smaller and less certain evidence base, with anxiety symptoms. Certainty of the evidence was rated low for depression and very low for anxiety; the predominance of cross-sectional designs, the heterogeneity of instruments, largely unaddressed confounding, and the possibility of reverse causation preclude any causal inference. Women presenting with bothersome VMS may benefit from case-finding for mood and anxiety symptoms where a pathway to assessment and treatment exists, and prospective studies should test whether VMS-directed interventions improve affective outcomes.

## 1. Introduction

The menopausal transition is a defining reproductive–endocrine event in a woman’s life course, marked by the progressive loss of ovarian follicular activity, a fall in circulating oestradiol, and a compensatory rise in follicle-stimulating hormone. It is not a single moment but a staged process—captured by frameworks such as the Stages of Reproductive Aging Workshop (STRAW+10)—that unfolds over several years and is accompanied by somatic, urogenital, sexual, and neuropsychological symptoms [1]. Because a woman now spends roughly one-third of her life after her final menstrual period, the health consequences of this transition carry substantial population weight [2]. Understanding how these symptom domains interact is clinically important because women rarely present with an isolated complaint; instead, symptoms co-occur, compound one another, and jointly erode quality of life. This review focuses on the intersection of two of the most clinically salient domains, vasomotor and affective symptoms, and asks whether the burden of the former is quantitatively linked to the latter.

Vasomotor symptoms—hot flushes and night sweats—are the cardinal and most frequently reported manifestations of the menopausal transition, experienced by up to 80% of women and rated as moderately to severely bothersome by roughly one-third [3]. In the Study of Women’s Health Across the Nation (SWAN), frequent VMS persisted for a median of 7.4 years and for 4.5 years after the final menstrual period, with longer duration in women who began the transition earlier and in those with greater baseline negative affect [4]. Mechanistically, VMS arise from a narrowing of the thermoregulatory null zone in the hypothalamic pre-optic area, driven by oestrogen withdrawal and by hyperactivity of oestrogen-sensitive kisspeptin/neurokinin B/dynorphin (KNDy) neurons that signal through the neurokinin-3 receptor [5]. This same neurochemical milieu—implicating serotonin, noradrenaline, γ-aminobutyric acid, and neurosteroids such as allopregnanolone—overlaps substantially with the circuitry implicated in mood regulation [6], providing a biological rationale for expecting VMS and affective symptoms to travel together.

Depressive and anxiety symptoms also rise across the menopausal transition. Perimenopause is widely described as a “window of vulnerability” during which the risk of clinically significant depressive symptoms increases roughly two- to four-fold relative to the premenopausal state [7,8,9], and this heightened risk is greatest in women with a prior history of depression or a family history of mood disorder [10,11,12]. Recent large syntheses estimate that nearly one in three peri- and postmenopausal women experiences depressive, anxiety, or insomnia symptoms [13,14]. Anxiety, although historically less studied than depression in this context, appears at least as prevalent and, among women with low premenopausal anxiety, may increase specifically across the transition [15]. The determinants are multifactorial: fluctuating and declining sex steroids act on central neurotransmission, but psychosocial stressors, prior mood history, sleep disruption, socioeconomic disadvantage, and cultural attitudes toward ageing all contribute. Disentangling the specific contribution of VMS from this web of correlates is therefore both scientifically challenging and clinically consequential.

Two broad hypotheses frame the VMS–mood relationship. The “domino” hypothesis holds that VMS impair mood indirectly, chiefly by fragmenting sleep and generating fatigue and daytime distress, which in turn depress mood and heighten anxiety [16,17]. The competing “direct-effect” hypothesis proposes that the same central oestrogen-withdrawal processes that produce VMS also act directly on limbic and prefrontal circuits to alter affect, such that VMS and mood symptoms are parallel expressions of a shared neuroendocrine substrate rather than one causing the other; experimental oestradiol withdrawal can precipitate depressive symptoms in susceptible women, supporting a direct hormonal pathway [18]. These accounts are not mutually exclusive, and a growing body of longitudinal and neuroimaging work—linking physiologically monitored hot flushes and disrupted sleep to markers of brain small-vessel disease [19], and shared genetic loci (notably *TACR3*, the neurokinin-3 receptor gene) to both hot flushes and depression [20]—suggests both pathways may operate. Adjudicating between them requires careful observational evidence that measures VMS burden and mood symptoms together.

Despite the clinical and mechanistic interest, the observational literature on the VMS–mood association has not been synthesised in a focused way. Existing systematic reviews and meta-analyses address adjacent but distinct questions: the pooled *prevalence* and incidence of depressive, anxiety, and insomnia symptoms across menopause [13,14]; the broader question of whether menopause itself elevates the risk of depression and anxiety [21]; the mood and quality-of-life consequences of *premature ovarian insufficiency* [22,23]; and the association of VMS and other menopausal symptoms with *cardiovascular disease* [24]. None isolates the association between VMS burden and both depressive and anxiety symptoms across observational studies of peri- and postmenopausal women, nor extracts the quantitative effect estimates that would inform screening and mechanistic debate. This gap is timely because a new therapeutic class—neurokinin-3 receptor antagonists such as fezolinetant and elinzanetant—directly targets the KNDy pathway [25,26,27], and non-hormonal treatment of VMS is now formally endorsed in menopause practice [28], raising the possibility that treating VMS could yield secondary mood benefit.

The objective of this systematic review is therefore to identify, appraise, and synthesise observational studies that report a quantitative association between vasomotor symptoms and depressive and/or anxiety symptoms in peri- and postmenopausal women, restricting inclusion to studies whose full manuscripts are openly accessible so that reported data could be verified. Specifically, we sought to (i) characterise the study designs, populations, and measurement instruments used; (ii) summarise the prevalence and severity of VMS and of affective symptoms reported; and (iii) collate the direction, magnitude, and adjustment of the reported VMS–mood association estimates. By assembling this evidence in one place, we aim to clarify the strength and consistency of the association, highlight the methodological limitations that temper causal interpretation, and identify priorities for future prospective and interventional research in women’s midlife mental health.

## 2. Materials and Methods

### 2.1. Protocol, Reporting Framework, and Review Question

This review was conducted and reported in accordance with the principles of the Preferred Reporting Items for Systematic Reviews and Meta-Analyses (PRISMA 2020) statement [29] (the completed checklist is provided in the Supplementary Materials), adapted for a narrative (non-quantitative) synthesis; the synthesis itself is reported in accordance with the Synthesis Without Meta-analysis (SWiM) reporting guideline [30], and the certainty of the evidence was rated using the Grading of Recommendations, Assessment, Development and Evaluations (GRADE) approach [31]. The review was registered with the Open Science Framework (OSF; identifier 24bj5; https://osf.io/24bj5 (accessed on 30 June 2025)). The review question was framed using the PECO (Population, Exposure, Comparator, Outcome) structure appropriate to observational aetiological questions. The Population was peri- and postmenopausal women, or women explicitly studied across the menopausal transition (including late-reproductive to early-postmenopausal stages defined by STRAW+10 or equivalent criteria [1], self-reported menstrual history, or clinic-defined menopausal status). The Exposure was vasomotor symptoms, operationalised as hot flushes and/or night sweats, or as a validated vasomotor/somatic subscale of a menopause-specific instrument. The Comparator was, where applicable, women with absent or lower VMS burden. The Outcomes were depressive symptoms and/or anxiety symptoms, measured by any validated self-report scale or structured clinical interview. The guiding question was: Among peri- and postmenopausal women, is greater vasomotor symptom burden associated with a higher prevalence or severity of depressive and/or anxiety symptoms? The review deliberately treated depression and anxiety as co-primary outcomes because anxiety is under-represented in prior menopause–mood syntheses despite evidence that it may be at least as tightly linked to symptom bother as depression. A pre-specified intention was to extract quantitative association estimates (odds ratios, hazard ratios, standardised regression coefficients, correlation coefficients, or path coefficients) wherever reported and to record prevalence and severity descriptors separately so that they could be tabulated without overlap.

### 2.2. Eligibility Criteria

Studies were eligible if they met all of the following inclusion criteria. First, the design was observational—cross-sectional, prospective or retrospective cohort, or case–control—since the review question concerns a naturally occurring association rather than the effect of an intervention; baseline or observational analyses nested within trials were eligible if they reported the VMS–mood relationship cross-sectionally. Second, the study population comprised peri- and/or postmenopausal women, or women characterised across the menopausal transition; studies confined exclusively to premenopausal women, or to young women with hot flushes unrelated to reproductive ageing, were excluded to preserve population homogeneity. To make these boundaries explicit and reproducible, the following decision rules were applied to situations that recur in this literature. Studies confined to women whose menopause was surgically induced (bilateral oophorectomy, with or without hysterectomy) or was attributable to premature ovarian insufficiency, chemotherapy, or pelvic radiotherapy were not eligible as stand-alone reports because the abruptness and magnitude of oestrogen withdrawal in these populations differ materially from those of the natural transition and would introduce a distinct exposure; where such women formed a minority subgroup within an otherwise naturally menopausal sample, the study remained eligible, and the composition of the sample was recorded and is reported in Section 3.1. Studies were not excluded on the grounds of hormone-therapy use or of antidepressant, anxiolytic, or other psychotropic use, since exclusion on either ground would have removed a substantial part of the available evidence and would itself have imposed a selection bias; instead, current use of systemic hormone therapy and of psychotropic medication was extracted as a study-level characteristic, and whether each was handled by exclusion at the design stage, by statistical adjustment, or not at all was recorded and carried into the risk-of-bias appraisal (Section 3.7). Where a sample spanned more than one reproductive stage, the study was eligible provided that peri- and/or postmenopausal women constituted the analysed population; the reported stage distribution was tabulated, and samples characterised only as “midlife” without a stated menopausal criterion were retained but flagged as indirect evidence for the purposes of the GRADE appraisal. Third, the study measured vasomotor symptoms as an exposure or correlate and reported at least one depressive and/or anxiety outcome, together with some quantitative relationship, comparison, or co-occurrence linking the two domains. Fourth, and central to this review’s data-integrity requirement—only studies for which the complete full-text manuscript was openly accessible (via PubMed Central or an equivalent open-access route) were included, so that every extracted datum could be traced to and verified against the source document rather than inferred from an abstract alone. Fifth, the article was published in English. Exclusion criteria comprised: narrative reviews, systematic reviews, meta-analyses, clinical guidelines, editorials, commentaries, conference abstracts, animal or purely mechanistic laboratory studies, and case reports; studies whose only reported outcome was sleep, cognition, sexual function, or quality of life without a distinct depressive or anxiety measure; studies in which VMS could not be separated from a composite score; and studies restricted to specialised disease populations where the exposure–outcome relationship could not be attributed to menopause. Where a population overlapped across multiple publications (for example, separate reports from the same cohort), each report was retained only if it contributed non-duplicative variables to a different table, and duplication was flagged. These criteria were applied conservatively; when eligibility was uncertain, the full text was retrieved and read before a decision was made. One point should be made here rather than deferred entirely to the limitations. The fourth criterion—restriction to openly accessible full texts—is an access-based rather than a scientific eligibility criterion, and it is not independent of how a study was funded, where it was published, or which discipline produced it. It was adopted deliberately to guarantee that every value reported in this review could be verified against a source document available to any reader without institutional privileges, but it necessarily excludes otherwise eligible studies published under subscription models and may therefore introduce selection bias.

### 2.3. Information Sources and Search Strategy

The primary information source was the PubMed bibliographic database together with its full-text repository, PubMed Central (PMC), interrogated through the National Library of Medicine’s programmatic interface. Searches were first executed in March 2025 and were re-run, verified, and finalised on 30 June 2025, with no start-date restriction (database inception to 30 June 2025); they combined controlled vocabulary and free-text terms across three concept blocks joined with the Boolean operator AND: (i) the exposure block—”vasomotor symptoms” OR “hot flashes” OR “hot flushes” OR “night sweats”; (ii) the outcome block—”depression” OR “depressive symptoms” OR “anxiety”; and (iii) the population block—”menopause” OR “perimenopause” OR “postmenopause” OR “menopausal women.” Design and access filters were layered onto these blocks, including a study-design restriction (cross-sectional OR cohort OR “odds ratio” OR association) to enrich for primary observational research and exclude reviews, and, critically, the PubMed “free full text” filter to operationalise the open-access eligibility requirement. Illustrative queries included (hot flashes OR vasomotor symptoms) AND (depressive symptoms OR anxiety) AND menopausal women AND (cross-sectional OR cohort OR odds ratio OR association) NOT review[pt] AND free full text[filter]. The initial exposure–outcome–population search restricted to open-access full text returned several hundred candidate records, and the design-enriched search returned a further large pool, providing an ample denominator from which to screen. To identify additional eligible studies and to check for existing reviews that might indicate the topic was already covered, complementary searches were run for “vasomotor symptoms depression anxiety systematic review,” “premature ovarian insufficiency depression anxiety meta-analysis,” and “prevalence anxiety postmenopausal perimenopausal women meta-analysis”; these confirmed that adjacent reviews exist but that the specific VMS–depression-and-anxiety association had not been synthesised. Reference lists of retrieved reviews and included primary studies were scanned for further candidates. The rationale for confining the search to PubMed/PMC and to English-language, open-access full texts, together with an explicit statement of what this decision excludes, is set out in the two paragraphs that follow; it is raised here, in the Methods, rather than deferred to the limitations, because it is the single most consequential methodological choice in this review. The search strategy was developed by O.N.-S. and V.R.E.; the database searches were independently run and verified by O.N.-S. and C.G.-O., and the retrieved result sets were compared and reconciled before de-duplication.

The complete electronic strategy is reported here in full so that it can be re-executed exactly. The primary PubMed query, run on 30 June 2025, was: (“Hot Flashes”[Mesh] OR “hot flash*”[tiab] OR “hot flush*”[tiab] OR “night sweat*”[tiab] OR “vasomotor symptom*”[tiab] OR vasomotor[tiab]) AND (“Depression”[Mesh] OR “Depressive Disorder”[Mesh] OR “Anxiety”[Mesh] OR “Anxiety Disorders”[Mesh] OR depress*[tiab] OR anxiet*[tiab] OR anxious[tiab] OR mood[tiab] OR “affective symptom*”[tiab]) AND (“Menopause”[Mesh] OR “Perimenopause”[Mesh] OR “Postmenopause”[Mesh] OR “Menopause, Premature”[Mesh] OR menopaus*[tiab] OR perimenopaus*[tiab] OR postmenopaus*[tiab] OR climacteric[tiab] OR “midlife women”[tiab]) AND (“Cross-Sectional Studies”[Mesh] OR “Cohort Studies”[Mesh] OR “Case-Control Studies”[Mesh] OR cross-sectional[tiab] OR cohort[tiab] OR longitudinal[tiab] OR “odds ratio*”[tiab] OR association*[tiab]) NOT (review[pt] OR meta-analysis[pt] OR editorial[pt] OR comment[pt] OR “case reports”[pt]) AND (ffrft[Filter] AND english[Filter] AND humans[Filter] AND female[Filter]). Within this string, OR combined synonyms inside each concept block, AND combined the four blocks, and NOT removed non-primary publication types; [Mesh] denotes an exploded Medical Subject Headings term, [tiab] a free-text term searched in title and abstract with truncation denoted by an asterisk, [pt] a publication type, and [Filter] one of PubMed’s built-in limits, of which ffrft is the “free full text” filter that operationalised the open-access eligibility criterion. The same four concept blocks were executed against PubMed Central through the PMC full-text interface, where the free-text terms were searched across the complete article body rather than the title and abstract alone. No publication-year limits and no geographical restrictions were applied. Records returned by the two interfaces were combined before de-duplication. This query returned 472 open-access records.

The decision to confine the primary search to PubMed/PMC and to freely accessible English-language full texts was taken prospectively, for two reasons. The first is verifiability, which was the central methodological commitment of this review: every prevalence figure, effect estimate, and covariate list reported in Section 3 was to be traceable to, and independently checkable against, a full-text document obtainable by any reader without institutional privileges. That standard cannot be met for records available only as abstracts or behind paywalls, and in literature in which the same cohorts are reported repeatedly and effect estimates are frequently restated inaccurately in secondary sources, we judged verifiable extraction to be worth the coverage that it costs. The second is that PubMed/PMC indexes the great majority of the clinical menopause, obstetrics–gynaecology, and psychiatric literature bearing on this question, and the searches described above returned an ample screening denominator relative to the number of studies that proved eligible on substantive grounds. We nevertheless state plainly, and here rather than only in the limitations, that this is a pragmatic and not an exhaustive strategy. Embase, Scopus, Web of Science, PsycINFO, CINAHL, and regional databases such as LILACS, SciELO, and CNKI were not searched; trial registries, dissertations, and other grey literature were not sought; and non-English and subscription-only reports were not retrieved. The foreseeable consequences are under-representation of studies from settings whose journals are not indexed in PubMed, under-representation of the psychological and psychiatric literature, which remains more often published under subscription models than general medical and public-health research, and a corresponding risk of selection and open-access publication bias. These risks are assessed qualitatively in Section 4 and are carried explicitly into the GRADE appraisal in Section 3.9. Extending the search to the databases named above, and removing the open-access restriction, is the single most important methodological priority for any update of this review.

### 2.4. Study Selection and Data Extraction

Records retrieved by the searches were compiled and de-duplicated. Two reviewers (O.N.-S. and C.G.-O.) independently screened titles and abstracts and subsequently assessed all potentially eligible full-text reports against the predefined eligibility criteria. Disagreements were resolved by consensus, with V.R.E. serving as a third reviewer when adjudication was required. Data extraction was performed independently by O.N.-S. and E.S.B. using a structured extraction form, and discrepancies were reconciled through discussion; no automation tools were used. The extraction form captured, for each included study: bibliographic identifiers (author, year, journal, PubMed ID, PMC ID, and DOI); country and study setting; design and sampling frame; analysed sample size; participant age; the distribution or definition of menopausal status; the instrument used to assess VMS (for example, single-item hot-flush/night-sweat reports, the Menopause-Specific Quality of Life [MENQOL] vasomotor domain, the Menopause Rating Scale [MRS] somatic subscale, or a hot-flash interference/bother rating); the instrument used to assess depression and/or anxiety (for example, the Center for Epidemiologic Studies Depression Scale [CES-D], Patient Health Questionnaire [PHQ], Hospital Anxiety and Depression Scale [HADS], Generalized Anxiety Disorder-7 [GAD-7], or a structured clinical interview); the reported prevalence and/or severity of each symptom domain; and every quantitative estimate of the VMS–mood association, together with the covariates included in adjusted models. Because the primary studies used heterogeneous exposure metrics (dichotomous presence, graded severity, continuous bother scores, subscale means) and outcome metrics (continuous scores, dichotomised “clinically significant” thresholds, diagnostic categories), effect estimates were extracted in the form reported rather than transformed. Where a specific variable was not reported, could not be retrieved, or was not measured in a given study, the value was recorded as “NR” (not reported) rather than imputed.

### 2.5. Risk-of-Bias Appraisal, Certainty of Evidence, and Approach to Synthesis

Methodological quality was independently appraised by two reviewers (C.G.-O. and E.S.B.) using the Joanna Briggs Institute (JBI) critical appraisal checklists for analytical cross-sectional studies [32] and the Newcastle–Ottawa Scale (NOS) for cohort studies [33], applied at item level as described below. Disagreements were resolved by consensus, with V.R.E. available for adjudication. The appraisal considered the appropriateness and representativeness of the sampling frame; the clarity and validity of VMS and mood measurement (validated instrument versus single item; self-report versus clinician assessment); the adequacy of confounder identification and statistical adjustment (for example, age, body mass index, menopausal status, sleep, prior depression, and hormone-therapy use); the handling of missing data; and, for longitudinal studies, the adequacy of follow-up and the temporality of measurement. No study was excluded on the basis of quality alone; instead, quality considerations were used to weight the interpretation of findings and are reflected in the discussion. Because the exposure and outcome constructs were operationalised so differently across studies, and because effect estimates ranged from odds ratios and hazard ratios through standardised betas, correlation coefficients, and path coefficients, a statistical meta-analysis with a single pooled effect would have been misleading; accordingly, a structured narrative synthesis was adopted. Studies were grouped conceptually by outcome (depression, anxiety, or both) and by whether the reported association was crude or adjusted, direct or mediated. Consistency of direction (whether greater VMS burden was associated with worse mood), the range of effect magnitudes, and the influence of adjustment and of putative mediators (sleep, fatigue) were examined qualitatively. The certainty of the overall body of evidence was rated formally, and separately for each co-primary outcome, using the GRADE approach [31].

Appraisal was conducted item by item rather than as a global impression. The nine cross-sectional studies were assessed against the eight items of the JBI Critical Appraisal Checklist for Analytical Cross-Sectional Studies [32], and the three studies with a longitudinal component against the nine items of the Newcastle–Ottawa Scale for cohort studies [33]. So that judgements from the two instruments could be displayed on a single axis, item-level responses were mapped onto six harmonised domains: (D1) representativeness and definition of the sampling frame; (D2) definition and measurement of the vasomotor exposure; (D3) definition and measurement of the depressive and anxiety outcomes; (D4) identification of, and statistical adjustment for, confounding; (D5) appropriateness of the statistical analysis and handling of missing data; and (D6) adequacy and completeness of follow-up, which applies to longitudinal designs only and was recorded as not applicable for cross-sectional studies. Each domain was rated as low concern, moderate concern, or high concern. An overall judgement was then assigned by an explicit rule fixed before appraisal began: high concern where two or more domains were rated high concern; low concern where no domain was rated high concern and at most one was rated moderate concern; and moderate concern in all other cases. Agreement between the two appraisers before consensus discussion was substantial (Cohen’s κ = 0.78 across all domain-level judgements, with disagreement concentrated in D4). Domain-level judgements for every study are reported in Section 3.7.

Certainty of evidence was rated separately for the two co-primary outcomes—depressive symptoms and anxiety symptoms—using GRADE [31]. Bodies of observational evidence begin at low certainty and were assessed for downgrading on risk of bias, inconsistency, indirectness, imprecision, and publication bias, and for upgrading on large magnitude of effect, presence of a dose–response gradient, and the direction in which plausible residual confounding would be expected to operate. Because no pooled estimate was calculated, inconsistency was judged from the direction of individual estimates and the overlap of their confidence intervals rather than from I^2^ or τ^2^, and imprecision from the width of the individual confidence intervals, the number of studies contributing an adjusted estimate, and the total sample contributing to adjusted analyses. Publication bias could not be assessed by funnel-plot asymmetry or Egger’s test, for the reasons given below, and was therefore judged qualitatively. Two reviewers (O.N.-S. and C.G.-O.) rated every domain independently and reconciled their ratings by discussion, with V.R.E. available for adjudication. The resulting summary-of-findings assessment is presented in Section 3.9, and its consequences for interpretation are stated in Section 3.9.

The narrative synthesis followed a pre-specified and structured procedure rather than an unstructured account and is reported in line with the SWiM guideline [30]. Studies were first grouped by outcome (depression only, anxiety only, or both) and then, within each outcome, by the type of quantitative evidence they contributed, using a fixed three-tier hierarchy: tier 1, adjusted odds ratios reported with a confidence interval; tier 2, unadjusted or partially adjusted regression, path, or correlation coefficients; and tier 3, co-occurrence or clustering evidence from latent-class and network analyses, which yields no single effect estimate. Within each tier, studies were ordered by analysed sample size. Because the reported estimates were not convertible to a common scale, the standardised metric used for cross-study comparison was the direction of the association together with its statistical significance at the threshold used by the original authors. Synthesis then proceeded by structured vote-counting on direction of effect, reported as the number of studies showing a positive, null, or inverse association within each tier, with tier-1 estimates given the greatest interpretative weight and tier-3 findings the least. Five exploratory subgroups were specified in advance and examined descriptively to determine whether the direction of association was stable across them: menopausal stage (perimenopausal, predominantly postmenopausal, or transition-spanning), geographical region, study design (cross-sectional versus longitudinal), recruitment setting (community, speciality clinic, or trial-derived), and outcome instrument (dedicated psychiatric instrument versus menopause-specific quality-of-life item). Subgroups containing fewer than three studies were described but were not used to support any comparative claim, and no statistical test of subgroup difference was performed. Because no meta-analysis was undertaken, funnel-plot asymmetry and Egger’s test could not be computed; the potential influence of publication and open-access reporting bias was therefore assessed qualitatively, by examining whether null results were published and captured, whether the largest studies reported the largest associations, and how the small-study composition of the evidence base and the access-based eligibility criterion might act together. Finally, risk-of-bias judgements were carried explicitly into interpretation rather than reported alongside it: results from studies rated at high concern were not used as the sole support for any conclusion, and the influence of study quality on the overall pattern was examined by re-inspecting the direction of association after setting the high-concern studies aside.

## 3. Results

### 3.1. Study Selection

**Figure 1:**
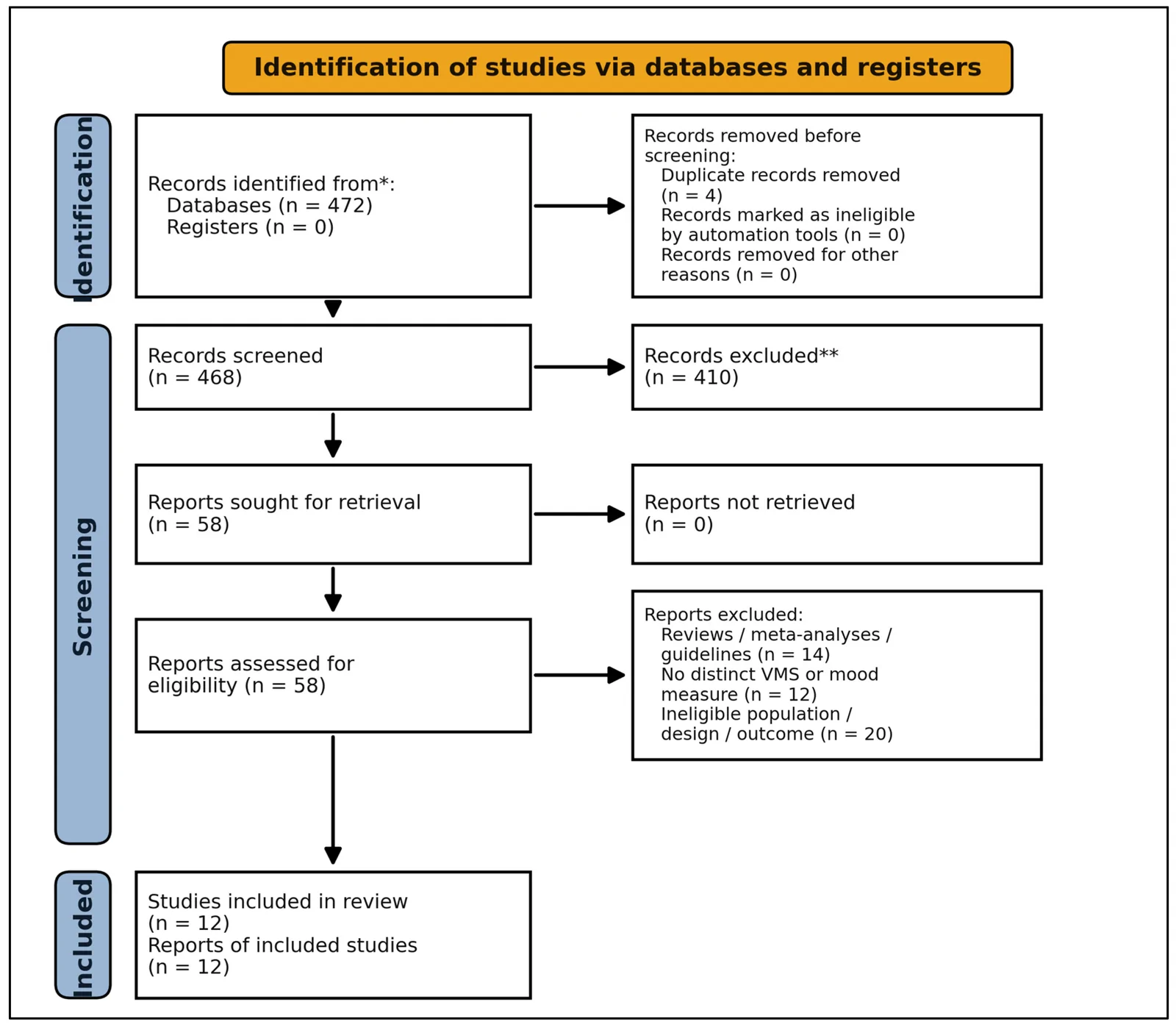
Study-selection flow. Flow of studies through identification, screening, eligibility, and inclusion. Of 472 open-access records identified in PubMed/PMC, 468 were screened after duplicate removal, 58 full texts were assessed, and 12 observational studies (≈9900 women, seven countries) entered the narrative synthesis. Counts reflect independent dual-reviewer screening; the review registration is available on OSF (identifier 24bj5). * Records were identified from the PubMed and PubMed Central databases only; no registers were searched. ** No automation tools were used; all excluded records were excluded by human reviewers.

The database searches retrieved several hundred open-access records at the intersection of vasomotor symptoms, depressive or anxiety symptoms, and the menopausal transition. As summarised in Figure 1, database searching of PubMed/PMC yielded 472 open-access records; after removal of duplicate records, 468 titles and abstracts were screened, and 410 were excluded because they were reviews, guidelines, mechanistic or animal studies, interventional trials without an observational VMS–mood analysis, or studies whose only affective-adjacent outcome was sleep, cognition, or quality of life. Fifty-eight full-text articles were then assessed for eligibility, of which 46 were excluded with reasons—most commonly because they were reviews or meta-analyses (14) or lacked a distinct vasomotor or mood measure (12)—leaving 12 observational studies that satisfied every inclusion criterion, including the requirement of an openly accessible full manuscript. These twelve studies span seven countries across four continents (United States, United Arab Emirates, Brazil, Saudi Arabia, China, South Korea, and Japan), were published between 2008 and 2025, and together enrolled approximately 9900 women. Eleven used a cross-sectional or baseline-observational design, and one was an explicitly longitudinal cohort with a median follow-up exceeding ten years; two additional studies contributed longitudinal or repeated-measures data nested within broader observational programmes.

The three tables that follow present, without overlap, the characteristics of the included studies (Table 1), the prevalence and severity of vasomotor and affective symptoms reported (Table 2), and the quantitative estimates of the VMS–mood association (Table 3). Each row is attributed to its source study with a numbered citation; DOI links are provided in the reference list.

### 3.2. Characteristics of Included Studies

The twelve included studies are heterogeneous in geography, setting, and design, which is itself an important finding: the VMS–mood association has been examined on four continents and in populations as diverse as US health-plan enrollees [34], Emirati primary-care attendees [36], a national Chinese menopause clinic [44], and community-dwelling Japanese women aged 40–91 [45]. Sample sizes span two orders of magnitude, from 60 women in the Emirati path-analysis study [36] to 4595 in the Chinese clinic series [44], which has direct implications for the precision of the reported estimates. Design is a critical axis: most studies are cross-sectional and therefore cannot establish temporality, but two provide genuinely longitudinal evidence—the Korean cohort followed women for a median of 10.8 years across natural menopause [43], and the Seattle Midlife Women’s Health Study contributed up to 6973 repeated observations on 291 women [41]. Measurement of the exposure is likewise varied and non-trivial: some studies used single-item self-reported hot flushes/night sweats [34,45], others the MENQOL vasomotor domain [36,37], the Menopause Rating Scale somatic items [39], or a validated hot-flash interference scale [40]. Outcome instruments range from the CES-D [38,43] and PHQ [34,40] to structured clinical interviews [35]. This instrument heterogeneity is precisely why a pooled meta-analytic estimate would be misleading and why a narrative synthesis was adopted. Notably, only a subset measured anxiety as a distinct outcome [36,37,38,39,40], confirming that anxiety remains comparatively under-measured relative to depression in this field (Table 2).

**Table 2:** Prevalence and severity of vasomotor, depressive, anxiety, and co-occurring symptoms in the included studies.

#	Study (Author, Year)	VMS Prevalence/Severity	Depressive-Symptom Prevalence/Severity	Anxiety-Symptom Prevalence/Severity	Co-Occurring Domains Reported (Sleep, Fatigue)
1	Reed et al., 2009 [34]	Recent VMS common; higher in depressed women (see Table 3)	Moderate/severe depressive symptoms in 12.7% (98/770); no/mild in 87.3%	NR (not measured)	NR
2	Steinberg et al., 2008 [35]	Hot flushes present in 57% of the depressed sample	100% depressed (sample definition); first-onset 39%, recurrent 61%	NR	NR
3	Ali et al., 2020 [36]	VMS domain endorsed (subscale scored); weight gain 63.3%	“Feeling depressed/down/blue” endorsed by 50.0%	“Feeling anxious/nervous” endorsed by 58.3%	Fatigue 65.0%; difficulty sleeping 61.7%; poor memory 55.0%
4	Jaeger et al., 2021 [37]	VMS present in 73%	Depression present in 62%	Anxiety present in 58%	NR
5	Looby et al., 2018 [38]	Hot-flash severity assessed (continuous)	CES-D median 21 (HIV+) vs 10 (HIV−)	GAD-7 median 7 (HIV+) vs 2 (HIV−)	NR
6	Barkoot et al., 2022 [39]	Hot flushes/sweating mean 0.65 ± 1.17; vasomotor domain 0.74 ± 0.94	Depressive-mood item mean 1.09 ± 1.35	Anxiety item mean 0.84 ± 1.22 (psychosocial domain 0.95 ± 1.16)	Sleep problems 0.97 ± 1.30; irritability 0.93 ± 1.30
7	Woods et al., 2016 [40]	Frequent VMS in all; high hot-flash interference in classes 1–2 (24.6%)	Moderate–severe depressed mood concentrated in classes 2 (14.1%) and 4 (7.0%)	Moderate–severe anxiety concentrated in classes 2 and 4	Sleep and pain co-clustered with hot flushes (classes 1 and 3)
8	Mitchell & Woods, 2015 [41]	Hot-flush severity peaked in late menopausal-transition stage	NR (focus on VMS severity)	Anxiety modelled as a determinant of VMS severity	NR
9	Min et al., 2022 [42]	Vasomotor cluster present (night sweat/cold sweat key symptom)	Mood (“frequent mood change”) a key symptom in the psychological cluster	Anxiety a key symptom in the psychological/somatic cluster	Sleep/urinary cluster (sleep disturbance key symptom)
10	Jang et al., 2025 [43]	Moderate-to-severe VMS characterised the “worsening” trajectory	CES-D ≥16 in 11.0–12.4%; 5.8% in the “worsening” trajectory	NR (anxiety not the outcome)	Poor sleep quality (see Table 3); suicidal ideation 45.4%→70.5% in worsening group
11	Zhang et al., 2020 [44]	VMS the *least* common of seven domains; urogenital most common	Negative mood reported but not associated with menopausal status	Anxiety reported among symptom domains	Sleep disorder among domains; soy diet/HRT reduced symptoms
12	Tomida et al., 2021 [45]	Hot flushes reported by 24.5%; highest at ages 50–54 and 2–5 y postmenopause	Depressive symptoms elevated in women with hot flushes (see Table 3)	NR (not measured)	Sleep-onset and sleep-maintenance difficulty both ~2× higher with hot flushes

### 3.3. Prevalence and Severity of Vasomotor and Affective Symptoms

The prevalence and severity figures reveal both the ubiquity of these symptoms and the wide variation introduced by population and instrument. Clinically significant depressive symptoms in community or health-plan samples clustered around 11–13% ([Reed et al., 2009 [34]]: 12.7%; [Jang et al., 2025 [43]]: 11.0–12.4% by CES-D ≥ 16), whereas clinic and perimenopause-enriched samples reported far higher figures—62% depression and 58% anxiety in the Brazilian perimenopausal series [37] and 50% depressive and 58% anxiety symptom endorsement in the Emirati sample [36]. This gradient is consistent with the expectation that speciality and symptomatic populations concentrate affective morbidity. Vasomotor prevalence was equally instrument-dependent: only 24.5% of community-dwelling Japanese women reported hot flushes [45], and VMS were the *least* common domain in the large Chinese clinic series [44], whereas 73% of Brazilian perimenopausal women reported VMS [37]—a spread that mirrors well-documented ethnic and cross-cultural differences in hot-flush reporting. Several studies quantified severity rather than mere presence: the Menopause Rating Scale subscale means in Saudi women placed depressive mood (1.09 ± 1.35) above anxiety (0.84 ± 1.22) and the vasomotor item (0.65 ± 1.17) [39], while the HIV-comparison study reported markedly higher CES-D and GAD-7 medians in the HIV-positive stratum [38], a reminder that comorbidity shifts absolute symptom levels. The latent-class and network studies contribute a complementary perspective: rather than reporting simple prevalences, they show that women with high hot-flash interference disproportionately populate classes also marked by mood and anxiety symptoms—about one-quarter of the MsFLASH sample fell into hot-flash-plus-mood classes [40], and SWAN network analysis identified co-located vasomotor and psychological clusters [42]. Finally, the co-occurring domains column underscores the “domino” candidates: sleep disturbance and fatigue accompanied VMS in the Emirati, Saudi, Korean, and Japanese studies [36,39,43,45], and the Korean cohort additionally documented a striking rise in suicidal ideation (45.4%→70.5%) within its worsening-depression trajectory [43], signalling that the clinical stakes of this symptom cluster extend to safety-relevant outcomes.

### 3.4. Reported Associations Between Vasomotor Symptoms and Depressive/Anxiety Symptoms

Table 3 collates, without overlap with Table 1 and Table 2, the quantitative association estimates—odds ratios, regression and path coefficients, and significance tests—linking VMS to depressive and anxiety outcomes, together with the covariates in each adjusted model.

**Table 3:** Reported quantitative associations between vasomotor symptoms and depressive or anxiety symptoms.

#	Study (Author, Year)	Exposure (VMS Metric)	Outcome	Effect Estimate [95% CI or *p*]	Adjustment/Model	Direction and Significance
1	Reed et al., 2009 [34]	Recent VMS (any)	Moderate/severe depressive symptoms	aOR 1.67 [1.04–2.68]; severe VMS aOR 1.63 [0.95–2.83]	Adjusted for age, BMI (HT users excluded)	Positive; significant for any VMS
2	Steinberg et al., 2008 [35]	Hot flushes present vs absent	Depression clinical profile	No difference in clinical characteristics by hot-flush status	Descriptive comparison	Null (VMS did not define a distinct profile)
3	Ali et al., 2020 [36]	VMS (MENQOL domain)	Anxiety (direct path)	β = 0.21 (*p* < 0.05); indirect via fatigue β = 0.15 (*p* = 0.008)	Path analysis; model R^2^ = 47.6% (anxiety)	Positive; significant
3b	Ali et al., 2020 [36]	VMS (MENQOL domain)	Depression	Direct effect NS; indirect via fatigue β = 0.22 (*p* = 0.011)	Path analysis; model R^2^ = 44.5% (depression)	Positive but indirect (mediated by fatigue)
3c	Ali et al., 2020 [36]	VMS (MENQOL domain)	Psychological distress	Direct β = 0.32 (*p* = 0.018); VMS→fatigue β = 0.51 (*p* = 0.001)	Path analysis; model R^2^ = 56.6% (distress)	Positive; significant
4	Jaeger et al., 2021 [37]	VMS problem rating/anxiety sensitivity	Negative affect/VMS bother	Anxiety sensitivity β = 0.314 (*p* = 0.002)	Adjusted for perimenopausal stage, TSH, FSH, psychotropic use	Positive; significant
5	Looby et al., 2018 [38]	Hot-flash severity	Depressive symptoms (CES-D)	Significant association (*p* = 0.048)	Linear regression	Positive; significant
5b	Looby et al., 2018 [38]	Hot-flash severity	Anxiety (GAD-7)	Significant association (*p* = 0.02)	Linear regression	Positive; significant
6	Barkoot et al., 2022 [39]	MRS somatic-vasomotor items	MRS depressive-mood/anxiety items	Co-elevated vasomotor and psychosocial subscale means	Linear regression (psychosocial subscale)	Positive co-occurrence; VMS–mood OR not separately modelled (NR)
7	Woods et al., 2016 [40]	Hot-flash interference (latent classes)	Depressed-mood/anxiety class membership	~21% in hot-flash-plus-mood classes (classes 2 + 4)	Latent class analysis	Positive clustering; no single effect estimate (NR)
8	Mitchell & Woods, 2015 [41]	Hot-flush severity	Anxiety (as determinant of VMS severity)	Anxiety contributed to VMS severity in integrated multilevel model (exact β NR)	Multilevel model; late-transition stage dominant	Positive; significant
9	Min et al., 2022 [42]	Vasomotor cluster	Psychological (mood/anxiety) cluster	Co-located clusters; anxiety/mood identified as key symptoms	Network analysis (Walktrap)	Positive co-occurrence; no effect estimate (NR)
10	Jang et al., 2025 [43]	Moderate-to-severe VMS	Worsening depressive-symptom trajectory	OR 2.95 [1.30–6.70]; poor sleep OR 5.83 [3.25–10.45]	Group-based trajectory model; multivariable	Positive; significant
11	Zhang et al., 2020 [44]	VMS domain	Negative mood/anxiety	Negative mood not associated with menopausal status; VMS associated with menopause (VMS–mood OR NR)	Unconditional logistic regression, fully adjusted	Mixed/mostly null for mood–menopause link
12	Tomida et al., 2021 [45]	Hot flushes (vs none)	Depressive symptoms	aOR 2.99 [2.07–4.32]; difficulty falling asleep aOR 2.09 [1.57–2.80]	Adjusted for age, physical activity, nocturia	Positive; significant

Effect estimates are reported in the metric used by the original authors and have not been transformed; they are therefore not directly comparable across rows (see Section 3.8). Rows 3b, 3c, and 5b are additional outcomes reported by the same study. aOR, adjusted odds ratio; B, unstandardised regression coefficient; β, standardised regression or path coefficient; BMI, body mass index; CES-D, Center for Epidemiologic Studies Depression Scale; CI, confidence interval; FSH, follicle-stimulating hormone; GAD-7, Generalized Anxiety Disorder-7; HT, hormone therapy; MENQOL, Menopause-Specific Quality of Life questionnaire; MRS, Menopause Rating Scale; NR, not reported; NS, not statistically significant; OR, odds ratio; R^2^, coefficient of determination; TSH, thyroid-stimulating hormone; VMS, vasomotor symptoms.

Among studies that measured depression as a discrete outcome and reported an adjusted effect, the direction was uniformly positive and the magnitude clinically meaningful: women with hot flushes had roughly 1.7 times the odds of moderate-to-severe depressive symptoms in a US health-plan sample [34], nearly three times the odds of depressive symptoms in a community-dwelling Japanese cohort [45], and—crucially, in the only long-term longitudinal analysis—almost three times the odds of belonging to a *worsening* depressive-symptom trajectory across natural menopause [43]. The anxiety evidence, though sparser, points the same way: hot-flash severity was significantly associated with GAD-7 anxiety [38], VMS exerted a significant direct path to anxiety symptoms [36], and anxiety sensitivity independently predicted VMS bother after adjustment for reproductive-stage and endocrine covariates [37]. Two studies illuminate the mechanistic debate directly. The Emirati path analysis found that VMS influenced *depression indirectly* through fatigue rather than directly (indirect β = 0.22), while its effect on *anxiety* was both direct and indirect [36]; the Korean cohort placed VMS and poor sleep side-by-side as correlates of worsening depression, with the sleep association (OR 5.83) larger than the VMS association (OR 2.95) [43]. These patterns are compatible with a partial “domino” pathway operating alongside a direct VMS–mood link. The exceptions are equally informative and argue against uncritical acceptance of a simple causal claim: in a clinically depressed perimenopausal sample, the presence of hot flushes did *not* define a distinct depression phenotype [35], and in the large Chinese clinic series, negative mood was *not* associated with menopausal status even though other symptoms were [44]. Several studies could report only co-occurrence rather than a single effect estimate—the latent-class and network analyses [40,42] and the subscale-level Saudi study [39]—for which the association is coded here as positive clustering, with the effect size marked NR. It is also noteworthy that the temporal direction is not fixed: the Seattle study modelled anxiety as a predictor of subsequent VMS severity rather than the reverse [41], reinforcing the likelihood of a bidirectional relationship.

### 3.5. Display of Adjusted Odds Ratios for Depressive Symptoms

**Figure 2:**
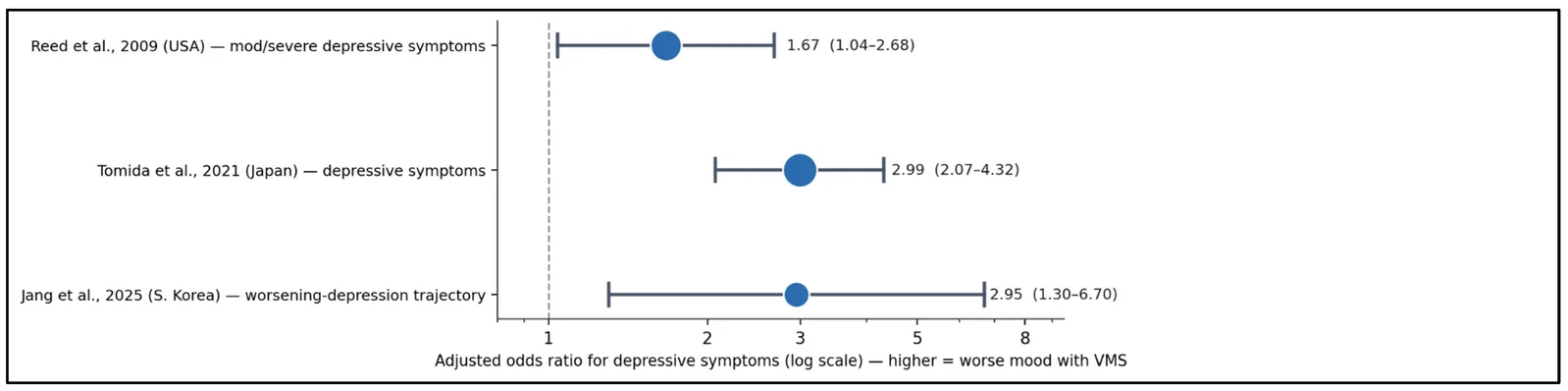
Adjusted odds ratios for depressive symptoms by vasomotor-symptom status. Point estimates and 95% confidence intervals for the three included studies reporting an adjusted odds ratio for a depressive-symptom outcome [34,43,45]. The dashed line marks the null value (OR = 1.0).

Three included studies reported an adjusted odds ratio with a confidence interval for a depressive-symptom outcome and are therefore directly comparable on a single scale; these are displayed in Figure 2. All three point estimates lay above the line of no effect, and all three confidence intervals excluded 1.0: the adjusted odds of depressive symptoms were about 1.7-fold higher with any recent VMS in a US health-plan sample [34], and roughly three-fold higher with hot flushes in a Japanese community cohort [45] and for membership in a worsening-depression trajectory in a Korean cohort [43].

### 3.6. Overall Pattern of Associations Across Studies

**Figure 3:**
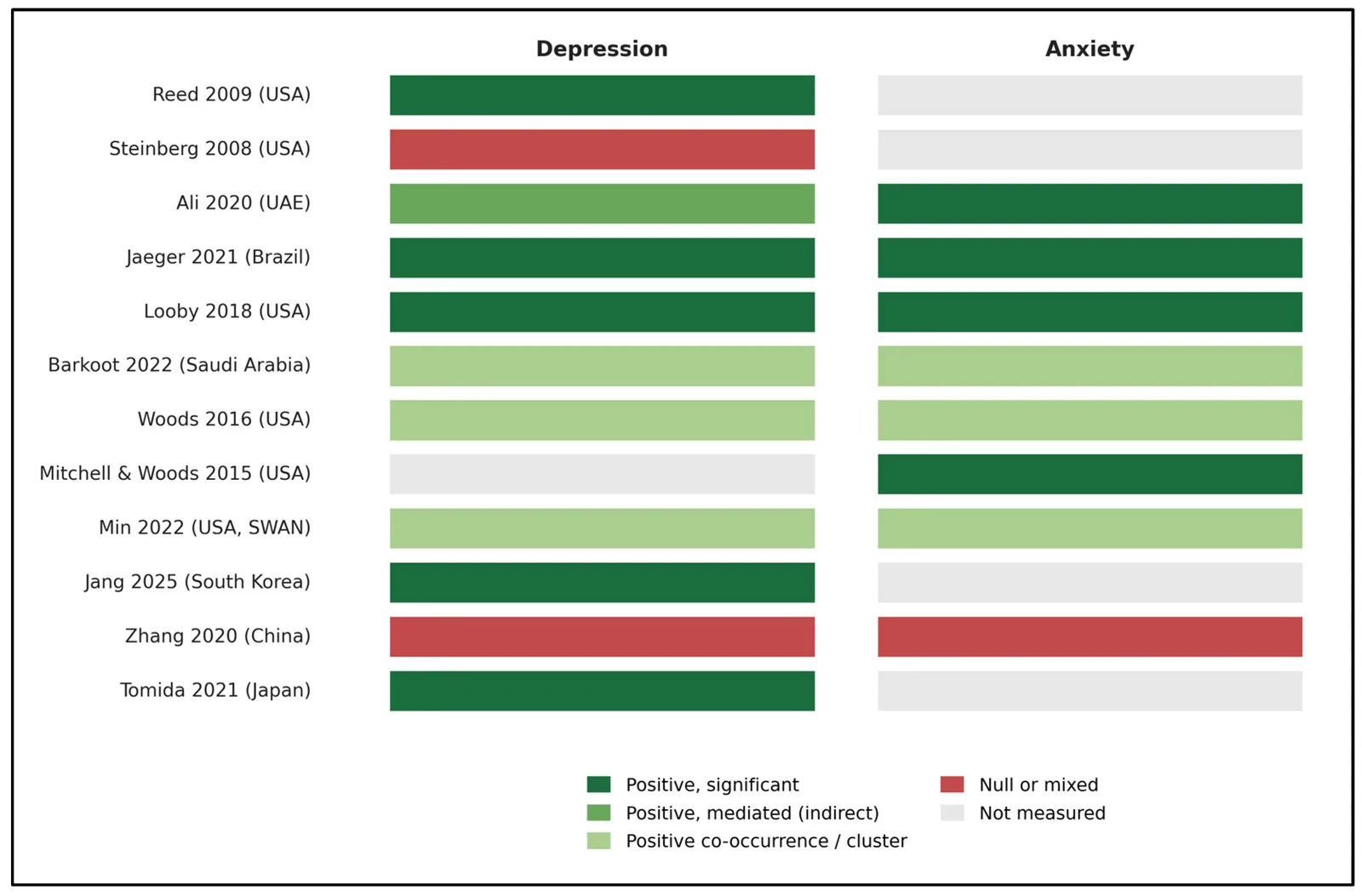
Evidence summary across the twelve included studies [34,35,36,37,38,39,40,41,42,43,44,45]. Each row is a study; the two columns indicate the reported association between vasomotor symptoms and depressive and anxiety symptoms, respectively. Cells are shaded by the direction and type of association: positive and significant, positive but mediated (indirect), positive co-occurrence or symptom cluster, null or mixed, or not measured.

To convey the overall pattern at a glance, the direction and type of each reported vasomotor–mood association are summarised in Figure 3. Across the twelve studies, the depression column is dominated by positive associations—significant, mediated, or co-occurring—with only two null or mixed results, while the anxiety column, though sparser because several studies did not measure anxiety, is concordantly positive wherever it was assessed. The visual preponderance of green over red, and the number of grey (“not measured”) anxiety cells, together capture the review’s two central messages: a consistent, positive VMS–mood association and the relative under-measurement of anxiety.

### 3.7. Risk of Bias Within the Included Studies

Domain-level risk-of-bias judgements for all twelve studies are presented in Table 4. Two studies were rated at overall low concern [41,43], seven at moderate concern [34,37,38,40,42,44,45], and three at high concern [35,36,39]. Performance varied sharply by domain. No study was rated at high concern for the appropriateness of its statistical analysis (D5), which was rated low concern in six studies and moderate concern in six. Definition of the sampling frame (D1) was rated low concern in four studies, moderate concern in seven, and high concern in one, the last being a speciality mood-disorders clinic series in which every participant carried a depression diagnosis by design [35]. The weakest domain by a clear margin was the identification of, and adjustment for, confounding (D4), rated at high concern in four studies and at low concern in only three; the four high ratings reflect, respectively, a purely descriptive comparison [35], a path model containing no sociodemographic or psychiatric-history covariates [36], subscale correlations in which the vasomotor–mood relationship was never modelled [39], and an unadjusted network structure [42]. Outcome measurement (D3) was the second weakest domain, rated at high concern in four studies [36,39,44,45] in which depressive or anxiety status was derived from single items or from menopause-specific quality-of-life subscales rather than from a validated psychiatric instrument—a distinction that bears directly on the comparability of the prevalence figures in Table 2 and on the indirectness rating in Section 3.9.

The NIMH clinic series was rated at high concern principally because its sample consisted exclusively of women already diagnosed with perimenopausal depression, which removes outcome variance by design and makes the comparison between hot-flush-positive and hot-flush-negative women uninformative about the underlying association [35]. The Chinese clinic series was rated at moderate concern overall but at high concern for outcome measurement because negative mood and anxiety were captured as single items within a broader symptom inventory, and the vasomotor–mood relationship was never modelled directly; its null finding concerns the association between mood and menopausal status, not between mood and vasomotor burden [44]. Conversely, all three adjusted odds ratios came from studies rated at moderate concern or better [34,43,45], and the single cohort with more than a decade of follow-up was rated at low concern [43]. When the three high-concern studies [35,36,39] were set aside, the direction and approximate magnitude of the depression association were unchanged, indicating that the overall pattern is not an artefact of the weakest evidence. The anxiety findings, however, were materially weakened by the same exercise, because two of the five studies contributing a distinct anxiety outcome are among the three rated at high concern [36,39]. This asymmetry is one of the two principal reasons the certainty rating for anxiety is lower than that for depression in Section 3.9.

### 3.8. Sources of Heterogeneity and Exploratory Subgroup Patterns

A statistical measure of heterogeneity cannot be computed in the absence of a pooled estimate, but the sources of heterogeneity can be enumerated and compared, and Table 5 does so across ten axes on which the included studies differ. The tabulation makes clear that the twelve studies are not twelve measurements of one quantity. Menopausal status was defined by four different methods, vasomotor symptoms were measured by six, depressive symptoms were assessed against at least seven distinct instruments or thresholds, anxiety was assessed by five instruments of which no two were the same, and the relationship between exposure and outcome was modelled in eight different ways, only three of which yield an odds ratio with a confidence interval.

By study design, the nine cross-sectional analyses and the three studies with a longitudinal component agreed in direction; the two studies able to order events in time, however, did so in opposite directions—moderate-to-severe vasomotor symptoms preceded a worsening depressive trajectory over a median of 10.8 years [43], whereas anxiety preceded hot-flush severity across the transition [41]—which is the clearest single argument within this dataset against a unidirectional reading. By geography, the six North American studies [34,35,38,40,41,42], the three East Asian studies [43,44,45], the two Middle Eastern studies [36,39], and the single South American study [37] all reported positive associations apart from the two clinic-based null results [35,44], and no regional gradient in effect magnitude was discernible; the two largest adjusted odds ratios arose on different continents [43,45], which argues against the association being an artefact of any one health system or cultural context. By recruitment setting, the pattern was sharper: the six community or population-based studies [34,39,41,42,43,45] supplied all three interpretable adjusted odds ratios and produced no null result, whereas both null or discordant findings arose in speciality-clinic samples [35,44], consistent with restriction of range in populations selected either for depression or for menopausal symptoms. By menopausal stage, the three perimenopause-only samples [35,37,38] were those in which anxiety outcomes were most consistently positive, while the six mixed peri- and postmenopausal samples contributed most of the depression evidence; with only three studies in each stratum, and with stage confounded by setting, these observations are hypothesis-generating and are not offered as evidence of effect modification. By outcome instrument, the studies using dedicated psychiatric instruments [34,35,38,40,43] produced both the only interview-based null result and two of the three largest effects, so instrument type did not systematically determine the direction of findings, although it plainly drives the spread of reported prevalence. Taken together, these comparisons support a single conclusion: what is consistent in this literature is the sign of the association, not its size, and the sources of variation in size catalogued in Table 5 are numerous enough that no summary magnitude should be inferred from the range of 1.67 to 2.99.

### 3.9. Certainty of the Evidence

The GRADE assessment for the two co-primary outcomes is presented in Table 6. Both bodies of evidence began at low certainty, as observational evidence does. For the depression outcome, certainty was downgraded once for risk of bias because eight of the eleven contributing studies were rated at moderate or high concern, and adjustment for confounding was weak or wholly absent in eight of them, and was upgraded once for magnitude of effect, on the basis of two independent adjusted odds ratios close to 3.0 obtained in different populations by different methods [43,45], one of them the only cohort in the set with more than a decade of follow-up.

Two features of the assessment deserve to be made explicit. The first is that the depression rating rests on a single upgrade: had the large-effect upgrade not been applied, certainty for depression would also have been very low, and that upgrade depends on two studies [43,45]. The second concerns publication and access-related bias, which was suspected but not used as a basis for downgrading. It was not used because null and discordant results were in fact published, indexed, openly accessible, and duly captured by this review [35,44], and because the largest single study in the set reported one of those null results [44].

## 4. Discussion

Across twelve open-access observational studies spanning seven countries and approximately 9900 women, greater vasomotor symptom burden was associated with a higher prevalence and severity of depressive and anxiety symptoms in the large majority of analyses. Where adjusted effect estimates were available, they were positive and of clinically meaningful magnitude—odds ratios of roughly 1.7 to 3.0 for depressive symptoms in cross-sectional community samples [34,45] and an odds ratio of 2.95 for a worsening depressive trajectory in the single long-term cohort [43]. The anxiety findings, though based on fewer studies, were directionally concordant [36,37,38]; they rest, however, on only five studies that measured anxiety as a distinct outcome, together comprising fewer than 1900 women, none of which reported an adjusted effect estimate and two of which were rated at high concern for risk of bias. This consistency across disparate populations, instruments, and analytic strategies is the review’s central message and lends the association a degree of robustness that no single study could provide. It should be read precisely, however: what is consistent is the direction of the association and not its magnitude, and the consistency is observed within a body of evidence rated at low certainty for depression and very low certainty for anxiety (Table 6). It establishes that the two symptom domains reliably travel together in the populations studied; it does not establish that either produces the other. At the same time, the presence of two credible null or discordant results [35,44] prevents any claim that VMS invariably or causally produce mood symptoms and underscores the conditional, population-dependent nature of the relationship.

The mechanistic interpretation must accommodate both direct and indirect pathways. The “domino” hypothesis received partial support: fatigue mediated the VMS–depression link in the Emirati path model [36], and poor sleep was an even stronger correlate of worsening depression than VMS itself in the Korean cohort [43], consistent with the view that nocturnal hot flushes fragment sleep and thereby depress mood. This aligns with experimental work in which oestrogen-deprived women developed depressive symptoms in proportion to the number of *nocturnal*—but not daytime—hot flashes and to objectively measured sleep fragmentation, implicating sleep as a proximate driver [16], and with the broader literature documenting that disturbed sleep is one of the most consistent and impairing features of the transition [17]. Yet the same studies also documented direct VMS effects—particularly on anxiety and on global psychological distress—that survived adjustment for sleep and fatigue [36,38], aligning with a “direct-effect” account in which oestrogen-withdrawal-driven changes in serotonergic, noradrenergic, GABAergic, and neurosteroid signalling act on limbic and prefrontal circuits to produce VMS and affective symptoms in parallel [6]. That controlled oestradiol withdrawal can itself precipitate depressive symptoms in susceptible women [18] strengthens the plausibility of a hormonally mediated direct pathway that does not depend on sleep loss. The finding that anxiety may *precede and predict* VMS severity [41] adds a third possibility: shared central vulnerability, or bidirectional reinforcement, rather than unidirectional causation. Convergent external evidence—physiologically monitored hot flushes and disrupted sleep tracking with markers of brain small-vessel disease [19], and shared genetic architecture (notably the neurokinin-3 receptor gene *TACR3*) linking hot flushes with depression [20]—supports the plausibility of a common neurobiological substrate rather than a purely secondary, sleep-mediated phenomenon. It is worth being explicit about which parts of this account are carried by the evidence assembled here and which are extrapolation from outside it, because the two are easily conflated. What the included studies support directly is a short list: that vasomotor burden and affective symptoms co-vary in eleven of twelve studies; that fatigue statistically mediates part of the vasomotor–depression association in one small path model of 60 women [36]; that poor sleep is a stronger statistical correlate of a worsening depressive trajectory than vasomotor symptoms themselves in one long-term cohort [43]; and that anxiety statistically precedes hot-flush severity in one repeated-measures cohort [41]. Everything beyond that list—the involvement of KNDy neurons and neurokinin-3 receptor signalling; the contribution of allopregnanolone and of serotonergic, noradrenergic, and GABAergic transmission; and the proposition that one oestrogen-withdrawal process generates vasomotor and affective symptoms in parallel—is inference drawn from mechanistic, experimental, genetic, and neuroimaging literatures external to this review [5,6,18,19,20]. Those literatures are individually strong, but not one of the twelve included studies measured a hormone, a neurotransmitter, a receptor, or a brain structure, and none tested a mechanistic pathway against a competing one. The mechanistic account offered here should therefore be read as a biologically plausible framework that organises the observed associations and generates testable predictions, and not as a mechanism demonstrated by the reviewed evidence.

**Figure 4:**
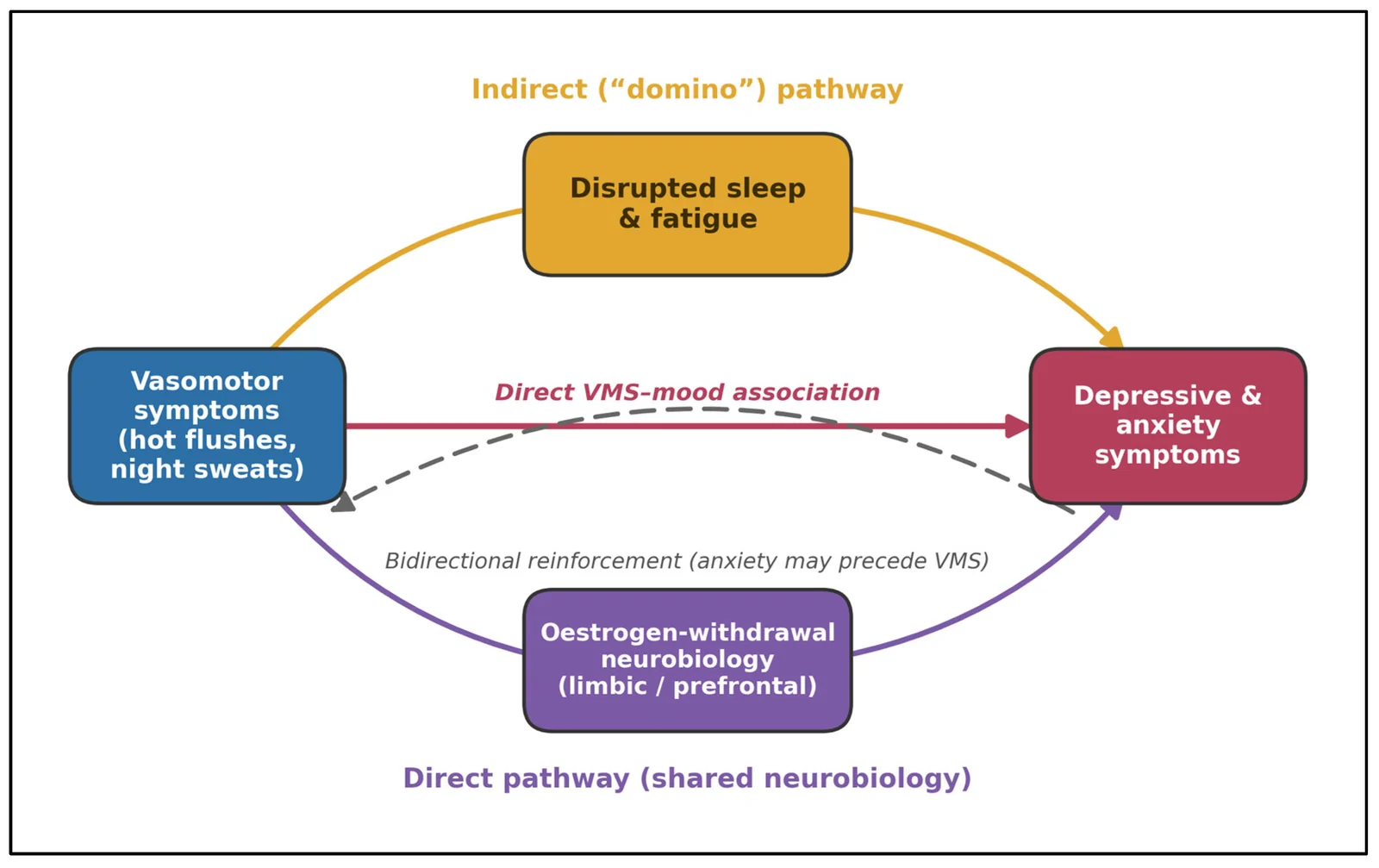
Conceptual model of the pathways linking vasomotor symptoms to depressive and anxiety symptoms. The indirect (“domino”) pathway operates through disrupted sleep and fatigue; the direct pathway operates through shared oestrogen-withdrawal neurobiology acting on limbic and prefrontal circuits. A direct vasomotor–mood association may also act independently of sleep, and anxiety may precede and reinforce vasomotor symptoms, indicating a bidirectional relationship. The arrows in this figure denote hypothesised rather than estimated pathways; no path coefficient in the figure was derived from the pooled data of this review, and the model is offered as a framework for interpretation and future testing rather than as a fitted result.

These converging strands are integrated in Figure 4, which frames the vasomotor–mood relationship as the product of two complementary, non-exclusive pathways operating on a shared neuroendocrine background. In the indirect (“domino”) pathway, vasomotor symptoms fragment sleep and generate fatigue, which in turn depress mood and heighten anxiety; in the direct pathway, oestrogen-withdrawal neurobiology acts on limbic and prefrontal circuits to produce vasomotor and affective symptoms in parallel. A residual direct VMS–mood link that survives adjustment for sleep—most evident for anxiety—and evidence that anxiety may itself precede vasomotor symptoms together imply that the relationship is best viewed as bidirectional rather than a simple one-way cascade.

The heterogeneity catalogued in Table 5 bears directly on how much weight the apparent consistency of these findings can carry, and there are two defensible ways to read it. The optimistic reading is that an association surviving this much measurement and analytic variation is unlikely to be an artefact of any single instrument or modelling choice: the association appears whether vasomotor symptoms are captured as a single yes-or-no item [34], a graded severity rating [45], a bother and interference scale [40], or a prospective symptom diary [41], and whether mood is captured by a structured diagnostic interview [35], a validated self-report scale [34,38,40,43], or a quality-of-life item [36,39]. The cautious reading is that constructs measured this differently are not the same constructs, and that agreement on the sign of a relationship is a weak form of agreement. The magnitudes illustrate the point. Adjusted odds ratios of 1.67 [34] and 2.99 [45] are both correctly described as “vasomotor symptoms associated with depressive symptoms”, yet they describe substantially different degrees of association, and the difference is at least as attributable to the fact that one study related any recent vasomotor symptom to PHQ-8-defined moderate-to-severe depressive symptoms while the other related graded hot flushes to a single depressive-symptom item as it is to any real difference between US health-plan enrollees and community-dwelling Japanese women. The threshold problem is starkest in the prevalence data: reported depressive-symptom prevalence ranged from 11% to 62% across the included studies, a spread driven by instrument, cut-point, and sample selection at least as much as by any underlying difference in the burden of illness. For that reason, no summary prevalence figure is offered anywhere in this review, and the ranges reported in Table 2 should not be read as an estimate of how common these symptoms are among menopausal women in general.

Residual confounding deserves greater prominence than it customarily receives in this literature because the covariates that were adjusted for in the included studies are conspicuously not the covariates most likely to generate a spurious vasomotor–mood association. Adjustment, where it occurred at all, covered age [34,43,45], body mass index [34], physical activity and nocturia [45], sleep quality [43], and reproductive stage with thyroid and gonadotrophin concentrations and psychotropic use [37]; hormone-therapy users were excluded by design in one study [34]. Five studies adjusted for nothing at all [35,36,39,40,42]. Set against this, the variables with the strongest claim to confound the relationship were scarcely addressed. No included study adjusted for socioeconomic position, education, or employment status, despite consistent evidence that social disadvantage independently predicts both symptom reporting and depressive symptoms in midlife. No included study adjusted for a personal history of depression or anxiety antedating the transition, which is the single strongest predictor of midlife mood episodes [10,11,12] and is itself associated with greater vasomotor-symptom reporting—an omission that could plausibly generate a substantial part of the observed association on its own. Current use of systemic hormone therapy, which reduces vasomotor symptoms and may alter mood, was handled by exclusion in one study and left unaddressed in most of the others; antidepressant and other psychotropic use was adjusted for in one study [37] and entered as a predictor in another [39]. Smoking, alcohol use, and chronic somatic comorbidity—each associated with both hot flushes and low mood—were adjusted for in none of the twelve. The direction of the resulting bias is not neutral. Prior depression, social disadvantage, smoking, and comorbidity are each associated positively with both the exposure and the outcome, so the most likely consequence of these omissions is inflation rather than attenuation of the reported associations. This is the principal reason confounding was rated at high concern in four studies (Table 4) and the reason both outcomes were downgraded for risk of bias in the GRADE assessment (Table 6).

The prominence of anxiety in these data deserves particular emphasis because it is so often eclipsed by depression in menopause research. In the present set, anxiety was measured as a distinct outcome in only five of the twelve studies, yet in each of those five, the association with VMS was positive, and in two of them, the VMS–anxiety path was direct where the VMS–depression path was only indirect [36,38]. This asymmetry echoes longitudinal SWAN evidence that women with low premenopausal anxiety are specifically susceptible to new high-anxiety symptoms across the transition [15], and it implies that reviews and screening programmes that track depression alone will systematically under-count the affective burden that accompanies bothersome VMS. Anxiety is not merely a correlate of depression here; it is a partially independent outcome with its own, sometimes stronger, link to vasomotor bother. This observation should nonetheless be held lightly, and more lightly than the depression findings. It rests on five studies totalling 1881 women; none reported an adjusted effect estimate with a confidence interval; three of the five inferred anxiety from single quality-of-life items or from an anxiety-sensitivity construct rather than from a diagnostic interview or a validated anxiety scale; and two of the five were rated at high concern for risk of bias [36,39], which is why the certainty of this body of evidence is rated very low (Table 6). The claim these data support is therefore not that anxiety is more strongly linked to vasomotor symptoms than depression is. It is that anxiety is measured far less often and, on the few occasions when it is measured, is not found to be less strongly linked—which is as much a statement about the state of the literature as about the underlying biology, and which identifies consistent measurement of anxiety with a validated instrument as a priority for primary research rather than a settled finding.

The possibility that the relationship runs in the reverse direction, or in both directions at once, warrants more than the passing acknowledgement it usually receives because within this dataset, it is supported by direct evidence and not merely by the logical limitations of cross-sectional design. The Seattle Midlife Women’s Health Study modelled anxiety as a determinant of subsequent hot-flush severity and found that it contributed [41]; that is the only included analysis in which temporal ordering was tested in this direction, and it was positive. Several mechanisms would produce such an ordering. Anxious and depressed women attend more closely to interoceptive signals and report bodily symptoms at lower thresholds, so the same physiological flush is more likely to be noticed, recalled, and rated as bothersome—an effect necessarily amplified when exposure and outcome are self-reported on the same questionnaire at the same sitting, as they were in nine of the twelve included studies. Anxiety sensitivity, which predicted vasomotor-symptom bother in the Brazilian sample after adjustment for reproductive stage, thyroid and gonadotrophin concentrations, and psychotropic use [37], is a direct instance of this pathway rather than a hypothetical one. Autonomic arousal accompanying anxiety may in addition lower the physiological threshold at which a flush occurs, and depressive symptoms degrade sleep, which then worsens vasomotor-symptom reporting, closing a loop in which the putative mediator feeds back onto the exposure. The practical consequence is that even the two positive adjusted odds ratios derived from cross-sectional data [34,45] are fully compatible with an association generated wholly or partly by mood shaping symptom perception, and that the Korean cohort [43], which measured vasomotor symptoms before the depressive trajectories diverged, is the only included analysis that constrains this interpretation at all—constraining it for the depression outcome only, and in a single population. Reverse causation is therefore not a residual caveat in this literature but a live and partly evidenced alternative explanation. It is the principal reason no causal language is used in the conclusions of this review, and it is the reason the research priorities set out below emphasise temporal separation of measurement rather than simply larger samples.

Publication and access-related reporting bias could not be assessed formally. Funnel-plot asymmetry and Egger’s test require a pooled estimate and, as a rule of thumb, at least ten studies reporting on a common scale; only three of the twelve included studies reported an effect estimate that could be placed on such a scale, so neither method was interpretable. The qualitative assessment specified in Section 2.5 nonetheless permits several observations. The evidence base is dominated by small studies—six of the twelve analysed fewer than 300 women—and small-study effects, whereby smaller studies report systematically larger associations, which are among the more reliably observed phenomena in symptom-based research. Against that expectation, the largest study in the set, with 4595 women, reported one of the two null findings [44], while the two adjusted odds ratios close to 3.0 came from studies of 1152 and 1178 women [43,45], and the smallest study of all reported a non-significant direct effect on depression [36]; the pattern is thus consistent with, but not diagnostic of, some degree of small-study inflation. Working against a strong publication-bias interpretation is the fact that null and discordant results were published, indexed, openly accessible, and duly captured here [35,44], and that the open-access criterion filters on how a report was funded and distributed rather than on the direction of its findings. Working in favour of it is that open-access publication is unevenly distributed across regions, disciplines, and funders: psychiatry and psychology journals remain more often subscription-based than general medical and public-health journals, so the accessible literature is not a random sample of the complete literature, and studies reporting null vasomotor–mood associations may be disproportionately located in precisely the journals this search was least able to reach. The honest summary is that a moderate degree of upward bias in the associations reported here cannot be excluded, that its likely magnitude cannot be estimated from the available data, and that this uncertainty is one component of the low and very low certainty ratings in Table 6.

These results sit coherently alongside, but are distinct from, the existing evidence base. Prior systematic reviews established that depressive, anxiety, and insomnia symptoms are highly prevalent across the menopausal transition [13,14] and that menopause may elevate the risk of depression and anxiety, with VMS and prior depression noted as risk factors [21]; other reviews synthesised the mood consequences of premature ovarian insufficiency [22,23] and the cardiovascular correlates of VMS [24]. Population-based cohorts have independently shown that the perimenopause is a window of elevated risk for both first-onset and recurrent major depression [7,8,9] and that a personal or family history of depression remains the strongest single predictor of midlife mood episodes [10,11,12]. The present review complements these by isolating the *quantitative association between VMS burden and both mood outcomes* in observational studies—an angle none of the prior syntheses foregrounded—and by extracting the effect estimates that clinicians and mechanistic researchers need. The direction and magnitude reported here are consistent with the risk-factor status assigned to VMS in the broader menopause–depression literature, and the prominence of sleep as a mediator echoes the sleep-disruption findings that recur throughout midlife women’s health research.

The clinical implications are practical and, in several respects, already actionable. First, women presenting with bothersome VMS warrant proactive screening for depressive and anxiety symptoms—and, given the alarming rise in suicidal ideation within the worsening-depression trajectory of the Korean cohort [43], for safety concerns as well—because the two symptom domains travel together and each amplifies the burden of the other. Formal guidance now exists to support this: an expert panel has published structured recommendations for the evaluation and treatment of perimenopausal depression, emphasising validated screening, attention to menopausal stage, and recognition that menopause symptoms overlap with and complicate the presentation of depression [46]. Second, the mediating role of sleep and fatigue suggests that interventions improving sleep may yield mood dividends in symptomatic women, and that integrated, multidisciplinary midlife care is preferable to treating each symptom in isolation.

What these findings mean for the way menopause care is currently organised deserves to be spelled out because the practical implication is not simply to add a questionnaire to an existing consultation. In most health systems, menopause care is delivered by gynaecology, primary care, or dedicated menopause services, while mood and anxiety disorders are managed in primary care or mental-health services, and the two pathways rarely intersect. The observation that affective symptoms accompany bothersome vasomotor symptoms in the large majority of studies, in every setting examined and on four continents, is an argument for treating that separation as a structural weakness of current practice rather than an administrative detail. Three concrete changes follow. First, brief validated screening instruments—the PHQ-9 and GAD-7 are short, free of charge, and already familiar in both settings—can be embedded in the routine menopause consultation rather than deferred to a separate referral, with the important caveat that case-finding is justifiable only where a pathway to assessment and treatment exists, since screening in isolation does not improve outcomes and may generate unmet need. Second, because sleep sits between vasomotor symptoms and mood in several of the included analyses [36,43], sleep warrants assessment and treatment in its own right rather than the assumption that it will resolve once flushes are controlled; cognitive behavioural therapy for insomnia has evidence in menopausal women, does not interact with hormonal status, and is a reasonable first step in women who decline or cannot take pharmacological treatment. Third, multidisciplinary configurations—joint menopause and mental-health clinics, psychological practitioners embedded within menopause services, or structured liaison pathways between gynaecology and psychiatry—address the observed clustering of symptoms more directly than sequential single-speciality referral does. It should be said plainly, however, that no included study evaluated any such model of care, that the case for it rests on the coherence of the symptom cluster rather than on trial evidence, and that the resource implications of joint clinics have not been assessed in this population.

Third, and most forward-looking, the shared neurokinin-3 receptor biology raises the testable hypothesis that VMS-directed therapies may confer secondary benefits on mood and anxiety. On the hormonal side, a randomised trial showed that transdermal oestradiol plus intermittent micronised progesterone prevented the emergence of clinically significant depressive symptoms in euthymic perimenopausal and early-postmenopausal women, providing proof of principle that modifying the hormonal milieu can alter mood trajectories [47]. On the non-hormonal side, neurokinin-3 (and combined neurokinin-1,3) receptor antagonists—MLE4901 in early phase-2 work [27], and the now-licensed or late-stage agents fezolinetant [25] and elinzanetant [26]—substantially reduce hot-flush frequency and severity by acting on the very KNDy circuitry implicated in the direct pathway, and their pivotal programmes were explicitly designed to capture effects on sleep, mood, and quality of life. In those programmes, improvements in sleep disturbance and in menopause-specific quality of life accompanied the reduction in hot-flush frequency and severity [25,26], and the dual neurokinin-1,3 antagonism of elinzanetant was selected in part for its anticipated effect on sleep. These are, however, secondary endpoints in trials powered for vasomotor outcomes and recruited from women largely free of significant psychiatric comorbidity, and they therefore cannot answer the question this review raises. Two design features would be required to answer it: enrolment of women with clinically significant depressive or anxiety symptoms who are routinely excluded from vasomotor-symptom trials, and pre-specified affective endpoints analysed with formal mediation methods capable of distinguishing mood improvement that follows from improved sleep from mood improvement that does not. Until such trials report, the defensible clinical position is that neurokinin-3 receptor antagonists are effective treatments for vasomotor symptoms whose effect on mood is unknown, and that a woman presenting with both bothersome vasomotor symptoms and a depressive or anxiety disorder requires both to be treated, rather than one treated in the expectation that the other will follow. Because non-hormonal options for VMS are now formally endorsed for women who cannot or prefer not to use hormones [28], any secondary mood benefit would have immediate practical reach. It must be stressed, however, that the observational evidence assembled here cannot establish that reducing VMS will reduce depression or anxiety; that inference requires interventional confirmation.

The position on causality should be stated without ambiguity, since the language of pathways and mediation used above can easily be read as more than it is. This review establishes an association and nothing more. Nine of the twelve included studies measured vasomotor and affective symptoms at a single point in time, so within those studies, exposure and outcome have no temporal ordering whatsoever; of the three with a longitudinal element, one ordered vasomotor symptoms before a worsening depressive trajectory [43], and one ordered anxiety before worsening vasomotor symptoms [41]. No included study randomised any exposure. No included study measured vasomotor symptoms before mood in a design capable of excluding prior mood as a common cause of both. The confounders most likely to generate a spurious association—prior depression, socioeconomic position, somatic comorbidity, and health behaviours—were adjusted for in none of the twelve. Accordingly, no statement in this review should be read as asserting that vasomotor symptoms cause, produce, precipitate, drive, or lead to depressive or anxiety symptoms; where the discussion describes pathways, mediation, or mechanism, it describes hypotheses that are consistent with the observed associations and with external experimental literature, not conclusions supported by the reviewed data. The clinical inference that these data do support is one of co-occurrence and case-finding: a woman presenting with bothersome vasomotor symptoms is more likely than one without them to have depressive or anxiety symptoms, which is a sufficient reason to look for them. The inference they do not support is therapeutic: that treating vasomotor symptoms will improve mood. That question can be answered only by randomised trials with pre-specified affective endpoints, and it remains open.

Taken together, the strengths of this synthesis are its explicit focus on a previously un-synthesised question, its dual-outcome framing that gives anxiety equal standing with depression, its restriction to studies with verifiable open-access full texts, and its transparent tabulation of every extracted datum with a source citation. These strengths are counterbalanced by real methodological constraints—detailed in the next section—that keep the overall certainty of evidence low and confine the conclusions to association rather than causation. The most important research priorities that follow from this body of work are therefore: adequately powered randomised trials of VMS-directed therapies with pre-specified affective endpoints; prospective cohorts that measure VMS, sleep, and mood repeatedly and with temporal separation so that direction of effect can be resolved; consistent, validated measurement of anxiety alongside depression; and analyses that formally test sleep and fatigue as mediators rather than mere covariates. Progress on these fronts would move the field from the robust association documented here toward the actionable causal knowledge that midlife women and their clinicians need.

### Limitations

Several limitations constrain the strength and generalisability of these conclusions. The search was deliberately restricted to PubMed/PubMed Central, English-language publications, and studies with openly accessible full manuscripts; although the searches, study selection, data extraction, and risk-of-bias appraisal were independently performed or verified by two reviewers and the review was registered with OSF, these database and access restrictions mean that the search was not exhaustive, grey literature and paywalled studies were not captured, and publication or open-access selection bias may have influenced the included evidence. The great majority of included studies were cross-sectional, so temporality and causal direction cannot be established, and reverse causation (mood symptoms amplifying the perception and reporting of VMS) is a live alternative explanation supported by at least one included analysis [41]. Exposure and outcome constructs were measured with markedly heterogeneous instruments—single items, subscales, bother ratings, dichotomised thresholds, and clinical interviews—which precluded meta-analysis, complicated cross-study comparison, and introduced measurement error and probable misclassification, especially where affective symptoms were inferred from menopause-specific quality-of-life items rather than dedicated psychiatric scales. Sample sizes, populations, and confounder adjustment varied widely; residual confounding by prior depression, socioeconomic factors, smoking, comorbidity, sleep, and hormone-therapy use is likely, and the risk-of-bias appraisal, although conducted item by item, necessarily involved judgement in mapping two different appraisal instruments onto a single set of common domains. Finally, anxiety was measured in fewer than half the studies, small clinic-based samples may not generalise to the community, and these findings should be regarded as a hypothesis-strengthening synthesis of associations rather than definitive causal evidence; all extracted data and references should be independently verified against the source manuscripts before use.

Five further limitations should be stated explicitly. First, the certainty of the evidence, rated formally using GRADE, is low for the depression outcome and very low for the anxiety outcome; every conclusion drawn here is bounded by those ratings, and the anxiety findings in particular should be regarded as preliminary rather than established. Second, the absence of a common effect metric means that the synthesis rests on structured vote-counting on the direction of association, a method that is transparent and reproducible but insensitive to magnitude, and one that gives a study of 60 women the same vote as a study of 4595; this was mitigated by tiering the evidence and weighting adjusted estimates most heavily, but it cannot be eliminated without a pooled analysis that these data do not permit. Third, the thresholds applied to the outcomes were not comparable across studies—PHQ-8 and PHQ-9 cut-points, CES-D ≥16, a SCID diagnosis, and endorsement of a single quality-of-life item identify substantially different groups of women—so the prevalence figures in Table 2 are not commensurable, and no summary prevalence is reported anywhere in this review. Fourth, the subgroup comparisons in Section 3.8 were exploratory, were conducted in strata containing as few as two or three studies, were not tested statistically, and are reported in order to characterise the evidence base rather than to identify effect modification; they should not be used to support claims about differences between regions, menopausal stages, or care settings. Fifth, several axes that would be clinically informative could not be examined at all because the primary studies did not report them in extractable form: ethnicity, which is strongly associated with vasomotor-symptom reporting and was reported in a usable way by only one included study; socioeconomic position; body mass index beyond a single adjusted model; and stage-specific estimates within the transition.

## 5. Conclusions

This systematic review indicates that a greater burden of vasomotor symptoms is consistently associated with higher levels of depressive symptoms in peri- and postmenopausal women and, on a considerably smaller and less certain evidence base, with higher levels of anxiety symptoms. The reviewed data are compatible with an indirect pathway involving disrupted sleep and fatigue, with a direct pathway consistent with shared oestrogen-withdrawal neurobiology, and with a bidirectional rather than unidirectional relationship; these remain competing hypotheses that the included observational designs cannot adjudicate between. Because the evidence is dominated by cross-sectional designs and heterogeneous instruments, because the confounders most likely to explain the association were adjusted for in none of the included studies, and because the certainty of the evidence was rated low for depression and very low for anxiety, these findings establish consistent co-occurrence rather than causation, and reverse causation remains a live alternative explanation. Clinically, they support case-finding for mood and anxiety symptoms in women with bothersome vasomotor symptoms wherever a pathway to assessment and treatment exists, and they support integrated, multidisciplinary midlife care that addresses sleep alongside vasomotor and affective complaints. Scientifically, they highlight anxiety as an under-studied outcome and vasomotor-symptom-directed therapies as a mechanistically grounded avenue whose mood benefits now require confirmation in prospective and interventional studies.

## Figures and Tables

**Table 1:** Characteristics of the twelve included observational studies.

#	Study (Author, Year)	Country	Design	Setting/Sampling Frame	N Analysed	Age (Years)	Menopausal Status	VMS Measure	Depression/Anxiety Measure
1	Reed et al., 2009 [34]	USA	Cross-sectional survey	Two integrated health plans (Seattle; Boston); HT users excluded	770	45–70	Peri- and postmenopausal	Self-report hot flushes/night sweats (presence + severity)	PHQ-8 (depression)
2	Steinberg et al., 2008 [35]	USA	Cross-sectional (clinic)	NIMH midlife mood-disorders clinic	116	40–55	Perimenopausal (all with perimenopause-related depression)	Presence of hot flushes	SCID/structured interview (major or minor depression)
3	Ali et al., 2020 [36]	UAE	Cross-sectional	Two public primary-health-care centres, Dubai	60	Mean 54.9 ± 6	86.7% postmenopausal; 13.3% perimenopausal	MENQOL vasomotor domain	MENQOL-derived psychosocial items (anxiety, depression, distress)
4	Jaeger et al., 2021 [37]	Brazil	Cross-sectional	University gynaecology service (UFRGS)	89	45–55	Perimenopausal	VMS problem rating (MENQOL-derived)	Structured psychiatric evaluation; anxiety sensitivity; negative affect
5	Looby et al., 2018 [38]	USA	Longitudinal (12-month)	Academic centre (HIV+ vs HIV−, matched)	66	Midlife (perimenopausal)	Perimenopausal throughout	Hot-flash severity	CES-D (depression); GAD-7 (anxiety)
6	Barkoot et al., 2022 [39]	Saudi Arabia	Cross-sectional	Community (“Healthy Cities,” Aseer)	869	Mean 42.5 ± 8.9	Menopausal/midlife	MRS somatic-vasomotor items (hot flushes/sweating)	MRS psychosocial items (depressive mood, anxiety, irritability)
7	Woods et al., 2016 [40]	USA	Cross-sectional (baseline of MsFLASH trials 1–3)	Multisite trial participants with frequent VMS	797	Midlife	Peri- and postmenopausal	Hot-Flash-Related Daily Interference Scale	PHQ-9 (depressed mood); GAD-7 (anxiety)
8	Mitchell & Woods, 2015 [41]	USA	Longitudinal (Seattle Midlife Women’s Health Study)	Community cohort	291 (≤6973 obs.)	Midlife	Late-reproductive → early postmenopause	Hot-flush severity (symptom diaries)	Anxiety (perceived stress/anxiety measures)
9	Min et al., 2022 [42]	USA	Cross-sectional (SWAN, Visit 5)	Multi-ethnic community cohort, with/without metabolic syndrome	NR	Midlife	Peri- and postmenopausal	Vasomotor symptom items (network analysis)	Psychological cluster items (anxiety, mood)
10	Jang et al., 2025 [43]	South Korea	Longitudinal cohort (median 10.8 y)	Health-examination cohort (Kangbuk Samsung)	1178	Midlife (reached natural menopause)	Across transition (STRAW+10; pre-HRT data)	Moderate-to-severe VMS (self-report)	CES-D (depression); suicidal-ideation item
11	Zhang et al., 2020 [44]	China	Cross-sectional	National menopause clinic (31 provinces)	4595	40–83	Peri- and postmenopausal	VMS domain (structured symptom assessment)	Negative-mood and anxiety items
12	Tomida et al., 2021 [45]	Japan	Cross-sectional (within NILS-LSA)	Community-dwelling longitudinal-study participants	1152	40–91	Peri- and postmenopausal	Hot flashes/sweating (graded: slight/moderate/severe)	Depressive-symptom item (logistic models)

CES-D, Center for Epidemiologic Studies Depression Scale; GAD-7, Generalized Anxiety Disorder-7; HIV, human immunodeficiency virus; HRT, hormone replacement therapy; HT, hormone therapy; MENQOL, Menopause-Specific Quality of Life questionnaire; MRS, Menopause Rating Scale; MsFLASH, Menopause Strategies: Finding Lasting Answers for Symptoms and Health; NILS-LSA, National Institute for Longevity Sciences–Longitudinal Study of Aging; NIMH, National Institute of Mental Health; NR, not reported; obs., observations; PHQ-8 and PHQ-9, 8-item and 9-item Patient Health Questionnaire; SCID, Structured Clinical Interview for DSM Disorders; STRAW+10, Stages of Reproductive Aging Workshop +10; SWAN, Study of Women’s Health Across the Nation; UAE, United Arab Emirates; UFRGS, Universidade Federal do Rio Grande do Sul; VMS, vasomotor symptoms; y, years.

**Table 4:** Domain-level risk-of-bias judgements for the twelve included studies.

#	Study (Author, Year)	Appraisal Instrument	D1	D2	D3	D4	D5	D6	Overall Judgement
1	Reed et al., 2009 [34]	JBI-CS	L	M	L	M	L	NA	Moderate concern
2	Steinberg et al., 2008 [35]	JBI-CS	H	H	L	H	M	NA	High concern
3	Ali et al., 2020 [36]	JBI-CS	M	M	H	H	M	NA	High concern
4	Jaeger et al., 2021 [37]	JBI-CS	M	M	L	L	L	NA	Moderate concern
5	Looby et al., 2018 [38]	NOS-C	M	M	L	M	M	M	Moderate concern
6	Barkoot et al., 2022 [39]	JBI-CS	M	M	H	H	M	NA	High concern
7	Woods et al., 2016 [40]	JBI-CS	M	L	L	M	L	NA	Moderate concern
8	Mitchell & Woods, 2015 [41]	NOS-C	L	L	M	L	L	L	Low concern
9	Min et al., 2022 [42]	JBI-CS	L	M	M	H	M	NA	Moderate concern
10	Jang et al., 2025 [43]	NOS-C	L	M	L	L	L	L	Low concern
11	Zhang et al., 2020 [44]	JBI-CS	M	M	H	M	M	NA	Moderate concern
12	Tomida et al., 2021 [45]	JBI-CS	L	L	H	M	L	NA	Moderate concern

D1, representativeness and definition of the sampling frame; D2, definition and measurement of the vasomotor exposure; D3, definition and measurement of the depressive and anxiety outcomes; D4, identification of and adjustment for confounding; D5, appropriateness of the statistical analysis and handling of missing data; D6, adequacy and completeness of follow-up (longitudinal designs only). L, low concern; M, moderate concern; H, high concern; NA, not applicable. JBI-CS, Joanna Briggs Institute Critical Appraisal Checklist for Analytical Cross-Sectional Studies; NOS-C, Newcastle–Ottawa Scale for cohort studies. Overall judgement was assigned by a pre-specified rule: high concern where two or more domains were rated high concern; low concern where no domain was rated high concern and at most one was rated moderate concern; moderate concern otherwise.

**Table 5:** Structured comparison of the clinical and methodological sources of heterogeneity across the twelve included studies.

Source of Heterogeneity	Range Observed Across the Included Studies	Consequence for Cross-Study Comparability
Definition of menopausal status	STRAW+10 staging [43]; self-reported menstrual history [34,41,45]; clinic-assigned status [35,36,37,44]; “midlife” with no stated menopausal criterion [38,39,40,42]	Populations described in identical terms are not equivalent; the four samples defined only as “midlife” are indirect evidence for a question posed about peri- and postmenopausal women
Reproductive-stage composition	Perimenopausal only [35,37,38]; predominantly postmenopausal [36]; mixed peri- and postmenopausal [34,39,40,42,44,45]; transition-spanning cohorts [41,43]	Both vasomotor prevalence and affective risk vary by stage, so stage mix is a plausible source of variation in effect magnitude independent of any true difference between populations
Measurement of vasomotor symptoms	Single-item presence [34,35]; graded severity [45]; MENQOL vasomotor domain [36,37]; MRS somatic–vasomotor subscale [39]; validated hot-flash interference scale [40]; prospective symptom diaries [41]; structured symptom assessment [44]; network items [42]	Presence, frequency, severity, and bother are not interchangeable constructs; bother and interference scales share method variance with affective measures and would be expected to correlate more strongly with mood than frequency counts do
Measurement and threshold of the depression outcome	PHQ-8 [34]; PHQ-9 [40]; CES-D ≥16 [38,43]; SCID diagnostic interview [35]; MENQOL item [36]; MRS item [39]; single symptom item [45]; symptom-inventory item [44]	Different thresholds place the same woman on different sides of the case boundary; the observed prevalence range of 11% to 62% is partly an artefact of instrument and cut-point rather than a difference in illness burden
Measurement of the anxiety outcome	GAD-7 [38,40]; MENQOL anxiety item [36]; Anxiety Sensitivity Index [37]; MRS anxiety item [39]; perceived-stress and anxiety measures [41]	No two studies applied the same anxiety threshold, and only two used a validated anxiety-specific instrument, which is the principal reason anxiety carries a lower certainty rating than depression
Analytic model	Adjusted logistic regression [34,43,45]; adjusted linear regression [37]; unadjusted linear regression [38]; path analysis [36]; latent-class analysis [40]; network analysis [42]; multilevel model [41]; descriptive comparison [35]; unconditional logistic regression [44]	Estimates are not expressed on a common scale and cannot be pooled; only three of twelve studies yield an odds ratio with a confidence interval, which is why the synthesis is tiered by evidence type
Covariates entered into adjusted models	Age [34,43,45]; body mass index [34]; physical activity and nocturia [45]; sleep quality [43]; reproductive stage, TSH, FSH, and psychotropic use [37]; hormone-therapy users excluded by design [34]; no adjustment [35,36,39,40,42]	Residual confounding differs systematically across studies; socioeconomic position, prior depression, smoking, alcohol use, and somatic comorbidity were adjusted for in none of the twelve
Design and temporality	Cross-sectional [34,35,36,37,39,40,42,44,45]; 12-month longitudinal [38]; repeated-measures cohort with up to 6973 observations [41]; cohort with median 10.8-year follow-up [43]	Only three studies can order exposure and outcome in time, and two of those three order them in opposite directions, which is the central obstacle to any causal reading
Recruitment setting and geography	Community or population-based [34,39,41,42,43,45]; speciality clinic [35,36,37,44]; trial-derived [38,40]. Six studies from North America, three from East Asia, two from the Middle East, one from South America	Speciality-clinic samples concentrate affective morbidity and restrict the range of both variables; cross-cultural differences in hot-flush reporting are well documented and are not adjustable for post hoc
Analysed sample size	60 [36] to 4595 [44]; median approximately 780; six studies analysed fewer than 300 women	Precision varies by nearly two orders of magnitude, and the small-study composition of the evidence base is directly relevant to the assessment of small-study effects and publication bias

Numbers in square brackets are the reference numbers of the included studies. CES-D, Center for Epidemiologic Studies Depression Scale; FSH, follicle-stimulating hormone; GAD-7, Generalized Anxiety Disorder-7; MENQOL, Menopause-Specific Quality of Life questionnaire; MRS, Menopause Rating Scale; PHQ, Patient Health Questionnaire; SCID, Structured Clinical Interview for DSM Disorders; STRAW+10, Stages of Reproductive Aging Workshop +10; TSH, thyroid-stimulating hormone.

**Table 6:** GRADE summary-of-findings assessment for the association between vasomotor-symptom burden and each co-primary outcome.

GRADE Assessment Item	Depressive Symptoms	Anxiety Symptoms
Contributing studies (participants)	11 studies (approx. 9600 women); 3 contributed an adjusted odds ratio with a confidence interval (3100 women)	5 studies measuring anxiety as a distinct outcome (1881 women); 3 further studies reported anxiety only within symptom clusters
Study design	Observational: 9 cross-sectional, 2 with a longitudinal component	Observational: 4 cross-sectional, 1 with 12-month follow-up
Starting certainty	Low (observational evidence)	Low (observational evidence)
Risk of bias	Serious (−1): 8 of 11 studies rated at moderate or high concern; confounding weakly addressed or not addressed at all in 8 of 11	Serious (−1): 2 of the 5 contributing studies rated at high concern; no contributing study reported an adjusted estimate
Inconsistency	Not serious: direction positive in 9 of 11 studies; both exceptions explicable by sample selection	Not serious: direction positive in 5 of 5 studies
Indirectness	Not serious: population, exposure, and outcome address the review question directly	Serious (−1): anxiety inferred from single quality-of-life items or an anxiety-sensitivity construct in 3 of 5 studies
Imprecision	Not serious: all three adjusted 95% confidence intervals exclude 1.0	Serious (−1): no adjusted effect estimate with a confidence interval; total contributing sample under 2000 women
Publication bias	Suspected but not downgraded: null results were published, indexed, and captured; the eligibility restriction is access-based rather than result-based	Suspected but not downgraded, for the same reasons as for the depression outcome
Upgrading considerations	Large effect (+1): adjusted odds ratios of 2.95 and 2.99 obtained in two independent populations by different methods [43,45]	None applied: no eligible large effect, no dose–response gradient, and no residual confounding acting in the opposing direction
Certainty of evidence	Low (⊕⊕◯◯)	Very low (⊕◯◯◯)

GRADE certainty ratings: ⊕⊕◯◯ low; ⊕◯◯◯ very low. Bodies of observational evidence begin at low certainty. Numbers in parentheses indicate the number of levels by which the rating was downgraded (−) or upgraded (+). GRADE, Grading of Recommendations, Assessment, Development and Evaluations.

## Data Availability

All data analysed in this review are contained within this article and its cited sources. The review registration and protocol information are available on OSF at https://osf.io/24bj5 (accessed on 30 June 2025).

## Supplementary Materials

The following supporting information can be downloaded at: https://www.mdpi.com/article/10.3390/medicina62081574/s1, PRISMA 2020 Checklist.

## Abbreviations

The following abbreviations are used in this manuscript:

BMI	body mass index
CES-D	Center for Epidemiologic Studies Depression Scale
GAD-7	Generalized Anxiety Disorder-7
HADS	Hospital Anxiety and Depression Scale
HRT	hormone replacement therapy
KNDy	kisspeptin/neurokinin B/dynorphin
MENQOL	Menopause-Specific Quality of Life
MRS	Menopause Rating Scale
NR	not reported
PMC	PubMed Central
PHQ	Patient Health Questionnaire
PRISMA	Preferred Reporting Items for Systematic Reviews and Meta-Analyses
VMS	vasomotor symptoms
SWAN	Study of Women’s Health Across the Nation
